# The Inflammatory Puzzle: Piecing together the Links between Neuroinflammation and Amyotrophic Lateral Sclerosis

**DOI:** 10.14336/AD.2023.0519

**Published:** 2024-02-01

**Authors:** Di He, Yan Xu, Mingsheng Liu, Liying Cui

**Affiliations:** Department of Neurology, Peking Union Medical College Hospital (PUMCH), Chinese Academy of Medical Sciences and Peking Union Medical College (CAMS & PUMC), Beijing, China

**Keywords:** amyotrophic lateral sclerosis, neuroinflammation, neurodegeneration

## Abstract

Amyotrophic lateral sclerosis (ALS) is a neurodegenerative disease that has a complex genetic basis. Through advancements in genetic screening, researchers have identified more than 40 mutant genes associated with ALS, some of which impact immune function. Neuroinflammation, with abnormal activation of immune cells and excessive production of inflammatory cytokines in the central nervous system, significantly contributes to the pathophysiology of ALS. In this review, we examine recent evidence on the involvement of ALS-associated mutant genes in immune dysregulation, with a specific focus on the cyclic GMP-AMP synthase (cGAS)-stimulator of interferon genes (STING) signaling pathway and N6-methyladenosine (m^6^A)-mediated immune regulation in the context of neurodegeneration. We also discuss the perturbation of immune cell homeostasis in both the central nervous system and peripheral tissues in ALS. Furthermore, we explore the advancements made in the emerging genetic and cell-based therapies for ALS. This review underscores the complex relationship between ALS and neuroinflammation, highlighting the potential to identify modifiable factors for therapeutic intervention. A deeper understanding of the connection between neuroinflammation and the risk of ALS is crucial for advancing effective treatments for this debilitating disorder.

## 1. Introduction

Amyotrophic Lateral Sclerosis (ALS) is a severe and fatal neurodegenerative disorder affecting the central nervous system (CNS), with a lifetime risk estimated at 1 in 350 individuals [1, 2]. The devastating disease is characterized by progressive neuronal degeneration, primarily impacting both upper and lower motor systems [3-5]. As the disease advances, patients experience a range of symptoms, including varying degrees of voluntary skeletal muscle weakness and atrophy, which result in impairments in limb movement, speech (dysarthria), swallowing (dysphagia), and eventually respiratory function [2, 6]. Despite the variable rate of disease progression, the majority of patients succumb to respiratory failure within approximately three years of symptom onset [7]. Additionally, a significant proportion of patients exhibit non-motor manifestations, such as cognitive impairment and behavioral changes [8], underscoring the multisystemic nature of ALS.

The genetic underpinnings of ALS are intricate, involving a combination of monogenic and oligogenic risks associated with pathogenic rare variants that exhibit substantial effect sizes [9]. To date, more than 40 genes have been implicated in ALS, with the most frequently mutated genes including chromosome 9 open reading frame 72 (*C9orf72*), superoxide dismutase 1 (*SOD1*), TAR DNA binding protein 43 (*TARDBP*) and fused in sarcoma (*FUS*) [9, 10]. Mounting evidence suggests that inflammatory mechanisms play a crucial role in the pathogenesis of ALS, particularly in the context of these highly penetrant genetic mutations. These mechanisms manifest as aberrant activation of immune cells, excessive production of inflammatory cytokines, and alterations in specific cellular populations [11, 12]. For instance, in *Sod1*^G93A^ mice, infiltration of CD8^+^ T cells into the CNS selectively triggers motor neuron loss by upregulating interferon (IFN)-γ production [13]. Therefore, the neuroinflammatory processes observed in ALS patients and disease models have gained recognition as a pivotal component of ALS pathophysiology.

The convergence of ALS-associated genes and aberrant inflammatory responses provides compelling evidence supporting the role of neuroinflammation as a significant contributor to the pathogenesis of the disease. However, the pathological evidence linking ALS and immune dysregulation suggests that neuroinflammation is an independent factor that promotes the development of the disease, extending beyond specific genetic causes. Traditionally, the activation of microglia and astrocytes is assessed through Iba1/CD86 and glial fibrillary acidic protein (GFAP) immunostaining, respectively, consistently showing a positive correlation with the rate of disease progression in ALS patients [14]. Histological observations have revealed morphological alterations in microglia, such as enlarged ramifications resembling tissue injury, in the motor cortex of individuals with sporadic ALS [12]. Additionally, prominent infiltration of peripheral immune cell populations has been observed in the parenchymal milieu of ALS patients and mice, indicating compromised blood-brain barrier (BBB) integrity [15]. Early immunohistochemical analysis of autopsy tissues has demonstrated a significantly higher presence of T cell infiltrates in ALS brain and spinal cord tissues compared to control specimens [16, 17]. Accordantly, increased expression of dendritic and monocytic transcripts has been observed in ALS spinal cords, which has been associated with more rapid disease progression [18].

In addition to changes in immune cell populations, altered cytokine production profiles have been observed even in apparently sporadic cases without identifiable mutations. The activation of proinflammatory monocytes/macrophages and a reduction in the levels of anti-inflammatory regulatory T (Treg) cells in peripheral blood have been directly correlated with disease progression [19, 20]. These correlations likely arise from significant alterations in circulating inflammatory cytokine levels, such as tumor necrosis factor (TNF)-α, interferon (IFN)γ, and interleukin (IL)-6 in ALS patients [21, 22]. Supporting this notion, RNA sequencing analysis of isolated ALS monocytes has revealed a distinct gene expression profile associated with inflammation, including IL-1B, IL-8, FOSB, and C-X-C Motif Chemokine Ligands (CXCLs) [23]. Furthermore, in vitro experiments using lipopolysaccharide (LPS)-stimulated dendritic cells derived from peripheral blood of ALS patients demonstrated significantly elevated levels of IL-8 and C-C Motif Chemokine Ligand 2 (CCL2) production [24]. Taken together, the current evidence suggests that a combination of motoneuron autonomous and immune-mediated non-cell autonomous mechanisms contribute to ALS neurodegeneration.

This review aims to provide comprehensive insights into the intricate interplay between ALS and neuroinflammation. It examines the most recent evidence regarding the involvement of ALS-associated mutant genes in immune dysregulation, focusing on the cyclic GMP-AMP synthase (cGAS)-stimulator of interferon genes (STING) signaling pathway and N6-methyladenosine (m^6^A)-mediated immune regulation in the context of neurodegeneration. The review delves into the aberrant phenotypes of immune cells, encompassing resident CNS glial cell activation (microglia and astrocytes), infiltration of peripheral immune cells (monocytes and macrophages), and impaired Treg cell functions. Moreover, the potential of understanding the relationship between neuroinflammation and ALS risk to identify modifiable factors for therapeutic development is explored. In the final section of the review, the prospects of emerging genetic and cell-based therapies are evaluated. By shedding light on the complex nature of the association between ALS and neuroinflammation, this review aims to inspire future research endeavors aimed at developing effective treatments for this devastating disorder.

## 2. Genetic connections between neuro-inflammation and ALS

### 2.1. ALS-implicated genes and ﻿cGAS/STING pathway

The use of genetic screening in ALS patients has resulted in the identification of numerous mutations, some of which affect immune gene function and contribute to neuroinflammation. Neuroinflammation in ALS is characterized by increased production of nuclear factor κB (NF-κB)-related cytokines [25] and activation of type I IFN signaling [26]. Under normal conditions, optineurin (OPTN) controls NF-κB activity, but its absence can lead to NF-κB translocation to the nucleus, triggering proinflammatory responses [27]. Similarly, angiogenin (ANG) can suppress TNF-α-induced inflammation by inhibiting TBK1-mediated NF-κB nuclear translocation [28]. Mutations in gene encoding sequestosome 1/p62 (SQSTM1) and valosin containing protein (VCP) also contribute to proteinopathies and immune dysregulation by impairing autophagy and the degradation of aggregated protein [29-31]. TANK-binding kinase 1 (TBK1) phosphorylates SQSTM1 and OPTN, and haploinsufficiency of TBK1 is associated with both ALS and frontotemporal dementia (FTD) [32].

Recent observations suggest that constitutive activation of STING pathways can cause neuro-inflammation and degeneration of dopaminergic neuron [33]. Yu et al. demonstrated that TDP-43 induces mitochondrial toxicity, leading to the release of mitochondrial DNA (mtDNA) into the cytoplasm. This activates the cytosolic cGAS/STING pathway and downstream type I IFN signaling [34, 35]. Microglia phagocytose cytoplasmic aggregates of TDP-43, a pathological hallmark observed in nearly all ALS and FTD cases [36], via the triggering receptor expressed on myeloid cells 2 (TREM2) [37]. Under physiological condition, TDP-43 is predominantly found in the nucleus, where it regulates RNA metabolism, but mutations in *TARDBP* enhance the propensity for TDP-43 aggregation [38]. Using induced pluripotent stem cell (iPSC)-derived motor neurons and TDP-43 mutant mice, the authors observed that ﻿pharmacological inhibition and genetic deletion of STING can mitigate TDP-43-induced neurodegeneration by downregulating ﻿inflammatory NF-kB and type I IFN gene expression [34].

**Figure 1.**
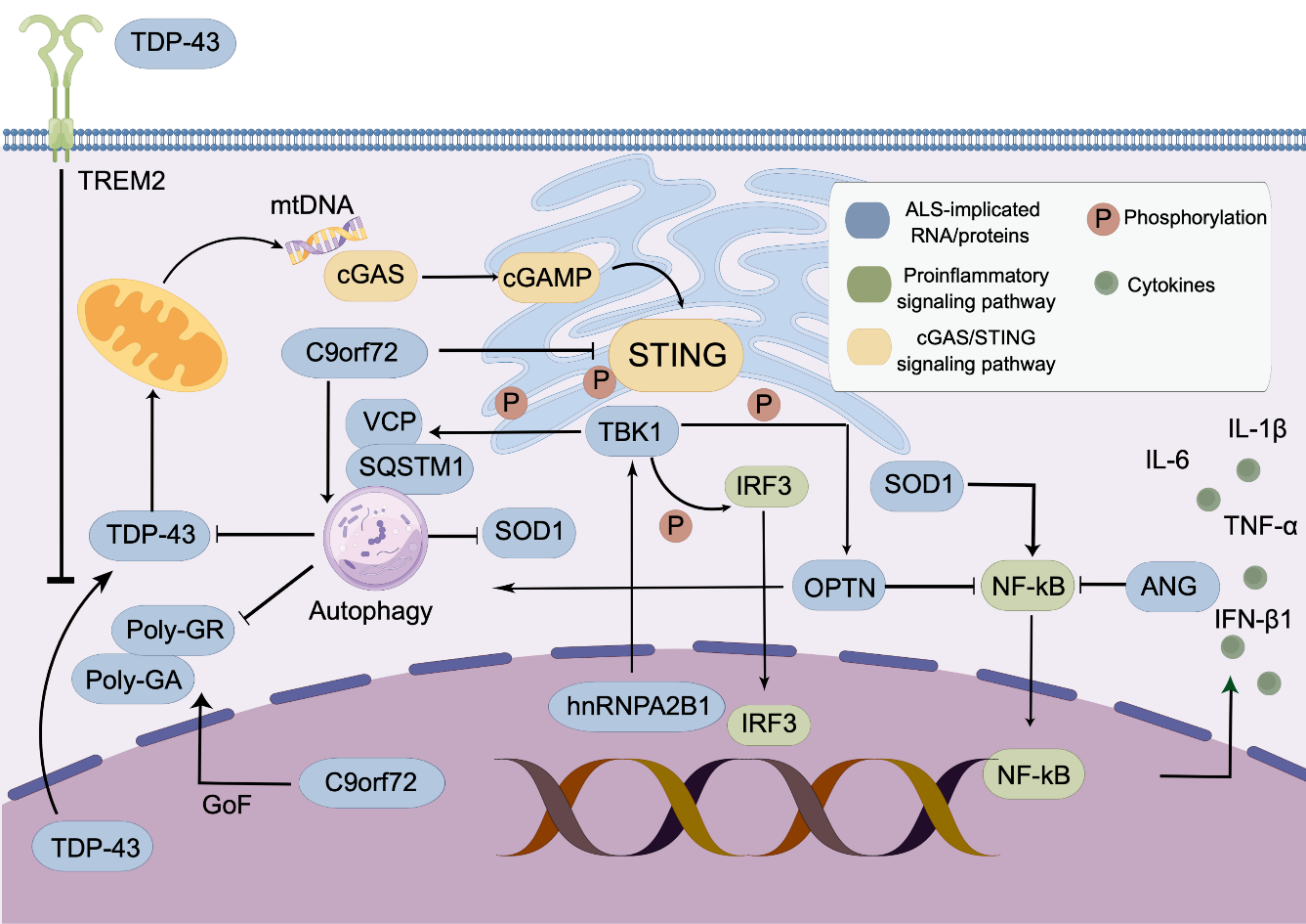
**cGAS-STING signaling pathway in amyotrophic lateral sclerosis (ALS)**. TDP-43 translocated into mitochondria induces the release of mitochondrial DNA, which is recognized by cGAS. The activation of cGAS enzymatic function leads to the synthesis of a potent ligand cGAMP, which further activates STING. This induces phosphorylation of STING and activation of downstream TBK1-IRF3 pathway. The cGAS-STING signaling pathway also promote NF-κB signaling, leading to hyperactive type I IFN responses. Multiple ALS-implicated genes are involved in this complex signaling network. This figure was created with Figdraw (ID: RWORS44333).

In addition to *TARDBP*, several ALS risk genes have been associated with proinflammatory activity mediated by cGAS/STING signaling. When cytoplasmic DNA binds to cGAS, it catalyzes the synthesis of cyclic GMP-AMP (cGAMP), which activates the adaptor protein STING. This leads to the recruitment and activation of TBK1 for the phosphorylation of interferon regulatory factor 3 (IRF3) [39-41]. C9orf72 regulates the degradation of STING through autophagy and the lysosomal pathway, while complete knockout of C9orf72 in mice sensitizes myeloid cells to STING and induces hyperactive type I IFN responses [42]. This finding was further supported by analyzing peripheral blood and CNS tissue from patients with C9orf72 repeat expansions, which revealed an elevated type I IFN signature that could be suppressed by STING inhibition [42]. STING also interacts with heterogeneous nuclear ribonucleoprotein A2B1 (hnRNPA2B1), which translocates to the cytoplasm upon detection of pathogenic DNA [43]. In cytoplasm, hnRNPA2B1 colocalizes with TBK1 and activates TBK1-IRF3 pathway to mediate IFN production [43]. Collectively, the fact that multiple ALS mutant genes alter cGAS/STING-related signaling provides evidence for the importance of this pathway in ALS pathogenesis (Fig. 1).

### 2.2. m^6^A-mediated regulation of inflammatory processes in ALS

Compromised RNA metabolism is a significant pathological feature of ALS, as evidenced by mutations in genes such as C9orf72, TARDBP, and FUS. These RNA-binding proteins (RBPs) have been found to be m^6^A-specific binding proteins, which regulate mRNA stability, splicing, and translation by recognizing m^6^A modifications [44-47]. Recent studies have highlighted the enrichment of m^6^A quantitative trait loci (m^6^A-QTL) in ALS postmortem brain and identified TARDBP as a potential m^6^A reader [48]. It has also been observed that TDP-43 preferentially binds to m^6^A-modified RNA, and ALS spinal cords exhibit widespread mRNA hypermethylation [49]. Our unpublished data indicates that a similar hypermethylated state can be observed in the peripheral blood of ALS patients, and the differentially methylated genes primarily influence biological processes associated with immune cell migration. Single-cell profiling of the ALS primary motor cortex and weighted gene co-expression network analysis (WGCNA) have indicated that several hub genes of the ALS-specific modules are associated with m^6^A RNA metabolism [50]. Given that transcripts encoding type I IFNs are heavily methylated and type I IFN-mediated inflammatory response plays a role in ALS neuroinflammation [51], it is likely that impaired mRNA metabolism and immune dysregulation converge in ALS pathophysiology.

The post-transcriptionally installed m^6^A modification is the most prevalent eukaryotic mRNA modification, which affects diverse biological processes via regulating mRNA splicing [52], export [53, 54], translation [55, 56], and degradation [57-59]. The functional consequences of RNA methylation are mediated by the coordinated action of m^6^A writers (methyltransferases), erasers (demethylases), and readers (m^6^A-specific binding proteins) [60-62]. For example, the ALS-implicated hnRNPA2B1 enhances TBK1-IRF3 signaling by blocking the recruitment of FTO and m^6^A removal from cGAS and STING transcripts, leading to enhanced production of IFNs [43]. RNA modifications also play critical roles in regulating immune cell biology [63]. Recent studies have demonstrated that m^6^A modification is involved in macrophage polarization, promoting proinflammatory M1 macrophages through pathways such as NF-κB and suppressor of cytokine signaling (SOCS) [64]. Deficiency of the methyltransferase METTL3 in macrophages reduces NF-κB pathway activity and TNF-α production, indicating a positive influence of RNA hypermethylation on M1 polarization [65, 66]. Other m^6^A regulators such as METTL14, ALKBH5, YTHDF1 and YTHDF2 have also been implicated in modulating type I IFN response [51, 67-69]. These findings suggest that m^6^A modification acts as a critical regulator of innate immunity potentially linked to ALS.

**Figure 2.**
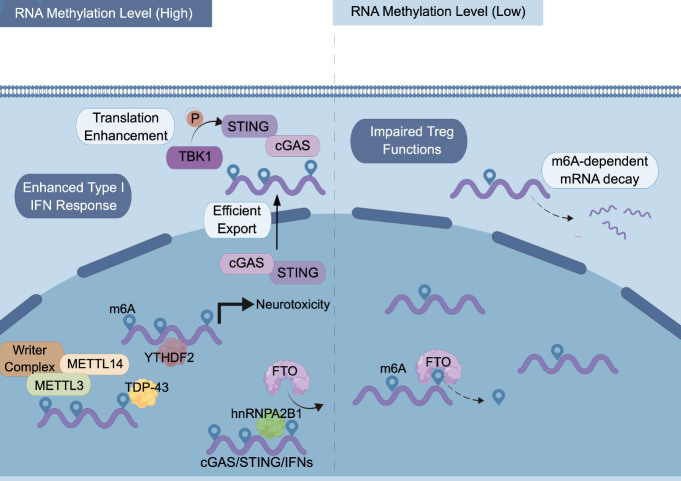
**﻿Molecular consequences of m^6^A modification in amyotrophic lateral sclerosis (ALS)**. m^6^A is post-transcriptionally installed by the writer complex consisting of METTL3, METTL14 and other accessory proteins. m^6^A can be removed by the m^6^A demethylases such as FTO, which can be blocked by the m^6^A reader hnRNPA2B1. TDP-43 preferentially binds to m^6^A-modified mRNA, while reduction of the m^6^A reader YTHDF2 mitigates TDP43-mediated. toxicity. m^6^A modification of mRNA transcripts leads to enhanced type I IFN-mediated inflammatory response and is involved in regulating Treg cell homeostasis. This figure was created with Figdraw (ID: AOUAR54dee).

The adaptive immunity associated with ALS pathophysiology is also regulated by m^6^A modification. Conditional deletion of *Mettl3* or *Mettl14* in murine CD4^+^ T cells impairs T cell differentiation and homeostasis [70, 71], while mice lacking *Mettl3* or *Mettl14* specifically in Treg cells displayed severe autoimmunity despite normal numbers of Foxp3^+^ Treg cells [72, 73]. The *SOCS* family genes, which negatively regulate inflammatory cytokine signaling by inhibiting IL-7 mediated STAT5 activation, are m^6^A-modified and exhibit slower mRNA degradation in T cells lacking *Mettl3* expression [71]. Because these *SOCS* genes are characterized by short half-life, m^6^A modification likely regulate adaptive immune response via affecting mRNA stability [74]. On the other hand, deficiency of the m^6^A eraser ALKBH5 results in attenuated CD4^+^ T cell responses to repress autoimmunity due to increased m^6^A modification on IFNγ and CXCL2 mRNA [75]. These findings indicate that m^6^A modification participates in multiple biological processes of immune cells, modulating inflammatory responses that may be relevant to ALS pathogenesis. Therefore, the mechanisms by which m^6^A regulates innate and adaptive immunity in ALS represent an emerging field for future investigation (Fig. 2).

### 2.3. C9orf72

The hexanucleotide repeat (G_4_C_2_) expansions in the noncoding region of *C9orf72* are the most common genetic cause of ALS [9, 76]. *C9orf72* is highly expressed in myeloid cell lines, indicating its involvement in immune responses [77-79]. Loss-of-function (LoF) mutations in *C9orf72* leads to severe autoimmunity and premature mortality in mouse models [80]. Mutant *C9orf72* has differential pathogenic effects on neuronal and non-neuronal cells. In *C9orf72* ^-/-^ mice, impaired autophagy function leads to a proinflammatory state in microglia with enhanced expression of IL-6 and IL-1β [81], although no motor neuron defects were observed [82]. Besides the LoF mechanism, evidence for a toxic gain-of-function (GoF) has also been observed in ALS patients with *C9orf72* mutations. *C9orf72* repeat expansions impair RNA metabolism by sequestering RNA binding proteins (RBPs) [83], while the dipeptide repeat proteins (DRPs) poly-GR and poly-GA generated by the expansions disrupt protein homeostasis and induce TDP-43 aggregation through TBK1 phosphorylation [84-86]. Additionally, poly-GA can activate the microglial inflammasome, leading to the production of IL-1β, which in turn induces TREM2 cleavage and inhibits phagocytosis of poly-GA aggregates [87]. These studies collectively suggest that altered functions of C9orf72 can directly influence myeloid cell immunity, which plays a critical role in ALS pathogenesis.

### 2.4 SOD1

Since its identification as a major ALS gene with a GoF mechanism [88], transgenic mice overexpressing human mutant SOD1 (mSOD1) have been extensively used for studying disease pathogenesis. Early investigations demonstrated that the neurotoxicity of mSOD1 is not cell autonomous to motor neurons [89], but rather depends on its expression in microglia [90]. This was further supported by experiments using chimeric mice, where wildtype motor neurons displayed ALS pathological features when surrounded by mSOD1-expressing glial cells, whereas wildtype glial cells significantly prolonged the survival of mSOD1-expressing neurons [91]. Mechanistically, the cytoplasmic aggregation of mSOD1 in microglia activates caspase-1 and IL-1β pathways, and inhibiting these pathways effectively attenuates inflammation and extends survival [92]. Microglia expressing mSOD1 also secrete higher levels of proinflammatory factors such as TNF-α, superoxide, and nitric oxide (NO) in response to LPS stimulation compared to wildtype microglia [93, 94], while selective inhibition of NF-κB in microglia rescues motor neurons from microglia-induced death [95]. In *SOD1^G93A^* mouse models, it has also been observed that loss of TBK1 kinase activity reduces the IFN response while aggravating SOD1 aggregation in motor neurons due to impaired autophagy function [96]. Based on current evidence, there is a strong correlation between the immune-driven mechanism and the neurotoxicity of mSOD1 in ALS, confirming the pivotal role of neuroinflammation in the pathogenesis of the disease.

## 3. Glial activation and immune cell infiltration

**Figure 3.**
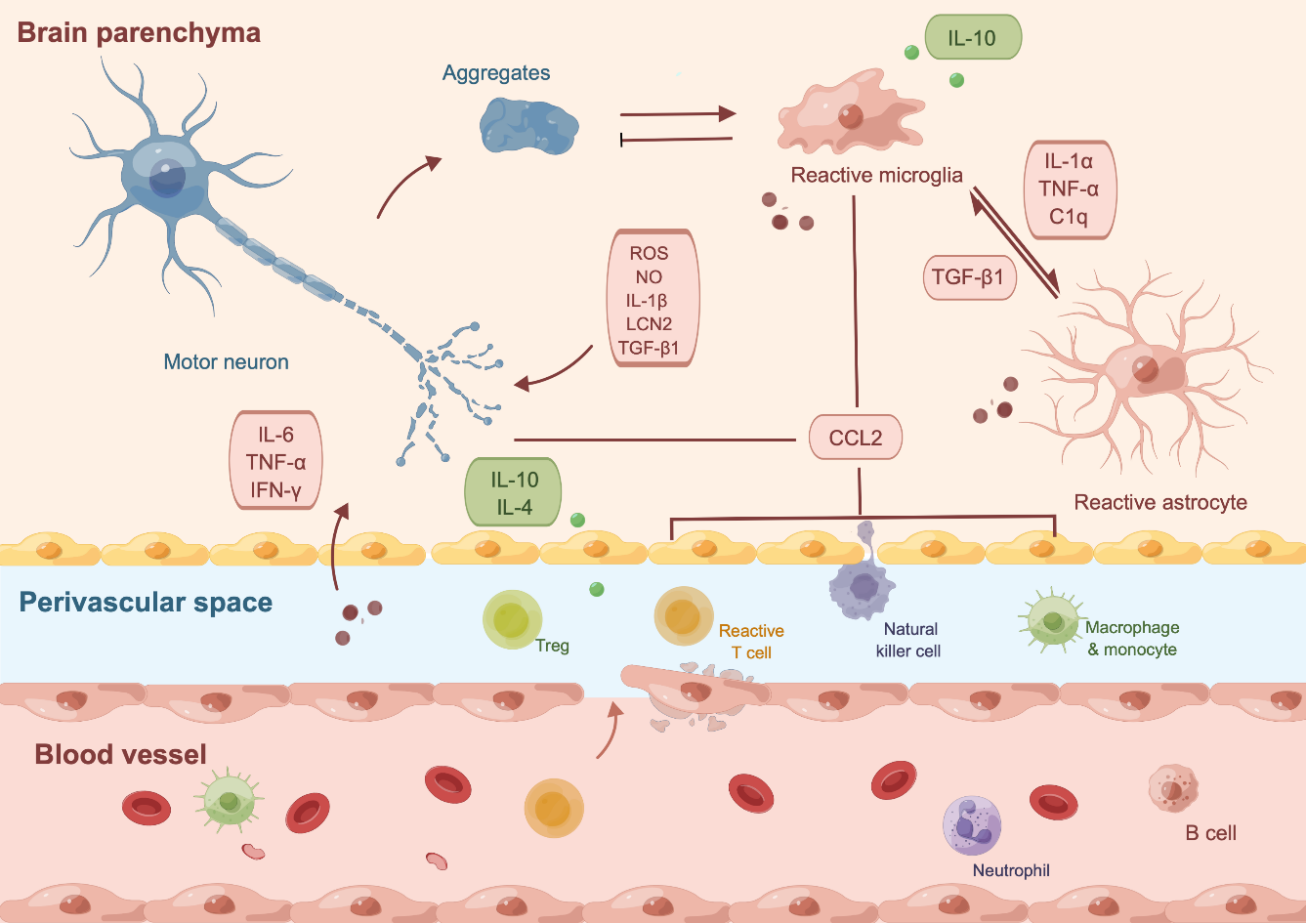
**Central and peripheral immune cells in amyotrophic lateral sclerosis (ALS)**. Protein aggregates from motor neurons induce reactive microglia, which further activate astrocytes to produce proinflammatory cytokines that have cytotoxic effects. Upregulated expression of CCL2 by microglia and neurons promote peripheral immune cell infiltration, which aggravates neuroinflammation. Additionally, various circulating peripheral immune cells traffic to the central nervous system following endothelial damage in patients with ALS. This figure was created with Figdraw (ID: IAUOIc7855).

ALS is traditionally considered a disease primarily affecting motor neurons. However, recent evidence has highlighted the crucial roles of CNS immune cells in both the onset and progression of the disease. Microgliosis and astrogliosis, characterized by the activation and proliferation of microglia and astrocytes, have been observed in the motor cortex of ALS patients and mice with TDP-43 pathology [97]. Transcriptomic analysis of postmortem ALS spinal cords has also demonstrated a significant increase in gene expression related to microglia and astrocytes, accompanied by a decrease in genes associated with oligodendrocytes and neurons, further highlighting the involvement of these CNS cell types in ALS-related neuroinflammation [98]. In the following sections, we will provide an overview of the key CNS cell types that contribute to neuroinflammation in ALS (Fig. 3).

### 3.1 Microglia

Microglia, which compromise approximately 5% of the glial cell population in the CNS, function as resident macrophages in the CNS [99, 100]. These cells constantly monitor their microenvironment and have the capacity to rapidly alter their morphology and gene expression profile in response to any perturbation in CNS homeostasis [100, 101]. Postmortem examinations have confirmed widespread microglial activation in ALS [102], and PET imaging has shown a correlation between microglial activation and disease progression [103, 104]. However, the conventional classification of microglial activation into two distinct phenotypes, namely the neurotoxic M1 phenotype and the neuroprotective M2 phenotype, is now considered overly simplistic [105]. Evidence suggests that the anti-inflammatory and pro-inflammatory responses exhibited by microglia should be viewed as a continuum between these polarized states, as demonstrated by the temporal shift from predominant expression of M2-associated markers at disease onset to M1-associated markers at end-stage disease [106].

During pre-symptomatic stages of ALS in a mutant SOD1 mouse model, microglia have shown an upregulated expression of the anti-inflammatory cytokine IL-10, and blockade of IL-10 has been found to significantly accelerate the clinical onset of the disease [107]. Additionally, studies have demonstrated that reactive microglia can selectively eliminate TDP-43 aggregates and promote motor function recovery in mouse models with reversibly induced neuronal TDP-43 aggregation [108]. Under normal physiological conditions, microglia typically eliminate TDP-43 via phagocytosis, whereas in its absence, a distinctive sequence of events that involves TDP-43 nucleocytoplasmic redistribution and neurodegeneration occurs, as observed in vivo using zebrafish spinal cord imaging [109].

As ALS progresses, degenerated neurons release proinflammatory substances that induce microglial activation, resulting in the expression of genes specific to neurodegeneration [110]. For example, exposure to exogenous mutant SOD1 recombinant protein has been shown to increase the release of free radicals and proinflammatory cytokines, mediated through toll-like receptor (TLR) 2, TLR4 and CD14 [111]. Both mutant SOD1 and TDP-43 can trigger microglial inflammasomes in a NLRP3-dependent manner, leading to caspase-1 activation and upregulation of IL-1β [112]. Overexpression of Poly(GA) in *C9orf72* mouse models can induce significant activation of microglial IFN responses, as well as selective neuron loss [113].

Taken together, current evidence suggests that microglia activation is critically involved in ALS-associated neuroinflammation. However, the extent to which microglial activation acts as a causative factor in accelerating neuronal loss or serves as a compensatory response to neurodegeneration remains incompletely understood [114, 115]. Further investigations are necessary to elucidate the dual functions of microglia at distinct stages of disease progression. Such studies would contribute our understanding of the pathogenic mutations responsible for ALS, including *SOD1*, *TDP-43* and *C9orf72*.

### 3.2 Astrocyte

Astrocytes, which constitute approximately 20% of glial cells in the CNS [99], play a vital role in maintaining the CNS microenvironment by providing trophic support to neurons, modulating synaptic activity, and facilitating repair processes [114, 116]. Similar to microglia, disruptions in CNS homeostasis can trigger astrocytic polarization towards the neurotoxic A1-like reactive phenotype [117]. However, current evidence does not support a neuroprotective role for astrocytes during the early stages of the disease. Instead, intracellular communication between astrocytes and other CNS cells often leads to astrocytic polarization towards the neurotoxic A1 phenotype. Notably, activated microglia release proinflammatory cytokines that promote the neurotoxic reactive state in astrocytes [118]. Deleting genes responsible for producing IL-1α, TNFα, and complement component C1q can alleviate this phenomenon, resulting in prolonged survival in the *SOD1^G93A^* mouse model [119]. Furthermore, selective expression of mutant SOD1^G86R^ in astrocytes results in normal morphology of motor neurons and microglia, despite evident signs of astrocytosis [120]. Co-culturing astrocytes overexpressing mutant TDP-43 with neurons expressing wild-type or mutant TDP-43 does not affect neuronal survival [121].

In contrast, reducing the expression of mutant *SOD1* in astrocytes has been shown to mitigate neurotoxicity mediated by patient-derived astrocytes in vitro [122]. This finding is further supported by studies demonstrating significantly delayed microglial activation and disease progression in mouse models [123, 124]. Transplanting wild-type astrocyte precursors has been found to ameliorate microgliosis and extend survival in *SOD1^G93A^* mice [125], whereas transplanting mutant *SOD1^G93A^* astrocyte precursors into wild-type mice leads to motor neuron degeneration [126]. The neurotoxicity of astrocytes likely depends on the release of astrocyte-derived soluble factors, such as transforming growth factor-β1 (TGF-β1), which interferes with the neuroprotective effects of microglia and T cells [127, 128]. Additionally, defects in energy metabolism (adenosine, fructose and glycogen) and disruptions in mitochondrial transport contribute to the neurotoxic properties exhibited by C9orf72-induced astrocytes [129, 130]. Moreover, astrocytes with restricted TDP-43 expression demonstrate downregulated expression of neurotrophic genes [131]. In transgenic mice with astrocyte-restricted expression of mutant TDP-43, motor neuron loss is accompanied by a progressive deficiency in the expression of glutamate transporter genes and induction of the neurotoxic lipocalin 2 (LCN2) in astrocytes [132]. Furthermore, iPSC-derived C9orf72 mutant astrocytes exhibit decreased secretion of antioxidant proteins and increased release of soluble factors associated with oxidative stress [133].

In summary, the pathogenic role of astrocytes in ALS primarily stems from their enhanced secretion of neurotoxic factors and reduced production of neurotrophic factors. Unlike microglia, astrocytes are not known to adopt a neuroprotective phenotype during the early stages of the disease. Instead, the activation of proinflammatory astrocytes depends on reciprocal communication with other CNS cells, particularly microglia, through the secretion of inflammatory mediators.

### 3.3 Immune cell infiltration

Recent research has revealed that during the development of ALS, various immune cells typically associated with the periphery infiltrate the CNS and join the resident glial cells [134]. Under normal circumstances, the influx of immune cells from the peripheral system into the CNS parenchyma is tightly regulated by specialized structures such as BBB. However, mutant SOD1-mediated endothelial damage occurs before the neurovascular inflammatory response and motor neuron death, suggesting that compromised BBB function contributes to the initiation of ALS-related neuroinflammation [135, 136]. Postmortem analyses of ALS tissues have confirmed widespread disruptions of vascular integrity in the choroid plexus epithelial layer and loss of pericytes around blood vessels [137]. Notably, heightened transcriptional activity of perivascular fibroblasts has been observed during the pre-symptomatic stage prior to microglial response in both ALS patients and mouse models, and the accumulation of the vascular cell marker protein SPP1 has shown a positive correlation with disease progression [138]. These findings underscore the potential therapeutic significance of targeting vascular dysfunction in ALS, as it may deter or even prevent the initiation of neuroinflammation and associated motor neuron death.

Apart from compromised BBB function, the entry of peripheral immune cells can also be mediated by increased expression of chemotactic proteins produced by resident CNS cells. For instance, elevated levels of CCL2 (also known as monocyte chemoattractant protein 1, MCP1) have been associated with the infiltration of macrophages in ALS spinal cord and cerebrospinal fluid (CSF) [18]. In ALS mouse models, splenic monocytes exhibit a polarized M1 signature with enhanced expression of the chemokine C-C motif receptor 2 (CCR2), while microglia show increased levels of CCL2 as the disease progresses, resulting in monocyte recruitment into the CNS [139, 140]. Furthermore, the infiltration of CCR2^+^ monocytes into the ALS motor cortex has been shown to occur through their interaction with the CCL2-expressing Betz cells, which typically do not express CCL2 [97].

However, there is contradictory evidence regarding the recruitment of peripheral myeloid cells into the CNS during disease development. While some studies have reported significant infiltration of peripheral myeloid cells into the CNS, others have demonstrated limited infiltration when an alternative method such as chemotherapy-induced myeloablation was used [141]. The discrepancy in these findings may be attributed to the possibility of artificial cell infiltration caused by irradiation-based myeloablation. Consequently, there are conflicting conclusions regarding whether the infiltration of peripheral myeloid cells in ALS is neurotoxic or neurotrophic [139, 142]. Therefore, further studies are needed to elucidate the effects of peripheral immune cell infiltration mediated by compromised BBB and CNS resident cells in ALS.

## 4 Peripheral immune cell regulation of CNS cells

Microglia are the primary resident immune cells of the CNS under normal conditions. However, it is important to note that the lymphatic system of the brain and CSF also harbor other types of immune cells that are distinct from those found in the circulating blood [143]. Postmortem analyses of ALS tissues have indicated the infiltration of macrophages/monocytes [18], CD4^+^/CD8^+^ T cells [17], and NK cells [144]. However, the role of peripheral immunity and its interaction with the CNS immune system in neurodegeneration is not as well understood as that of the residential glial cells within the CNS. In the subsequent sections, we will examine the existing evidence concerning the potential involvement of peripheral immune cells in ALS pathogenesis.

### 4.1 Macrophage/monocyte

ALS patients exhibit a proinflammatory transcriptomic profile in circulating monocytes, particularly in those with rapid disease progression [23]. Specifically, monocytes derived from ALS patients show a higher tendency to differentiate into a pro-inflammatory M1 phenotype, characterized by increased expression of IL-6 and TNF-α, compared to healthy controls [145]. The loss of *C9orf72* in myeloid cells hinders STING protein degradation via the autolysosomal pathway, leading to hyperactive type I IFN responses and autoimmunity [42]. In vitro studies indicate that IFNγ-stimulated macrophages secrete inflammatory mediators that further stimulate microglia to produce TNF and NO [146]. In addition, conditioned medium from M1 macrophages induces a reactive gene expression in astrocytes [147]. Collectively, these findings suggest that peripheral myeloid cells carrying ALS-implicated mutations can influence microglial functions during motor neuron degeneration, providing a proof-of-concept.

Research has also shown potential benefits of periphery-derived myeloid cells. Activated macrophages have been observed to infiltrate the peripheral nervous system (PNS) of mutant *SOD1* mice, constituting a distinct population from resident microglia in the spinal cord [148]. The infiltration of monocytes into the CNS has been positively associated with delayed disease onset and improved motor neuron survival in vivo, suggesting a protective role for peripheral monocyte infiltration during the early stages of the disease [142]. It has been proposed that infiltrating macrophages may act as regulatory agents, suppressing microglia-mediated neuro-inflammation [149]. Furthermore, damaged motor neurons actively recruit peripheral macrophages and CD8^+^ T cells by upregulating their expression of CCL2, major histocompatibility complex class I (MHCI), and C3, which, in turn, delays muscle denervation in SOD1 mice with slow disease progression [150]. These findings indicate the potential neuroprotective effects of periphery-derived myeloid cells in the context of ALS pathology.

In a recent study by Chiot *et al*., the roles of peripheral macrophages were investigated by replacing mutant macrophages with genetically modified macrophages that exhibit reduced neurotoxic reactive oxygen species (ROS) responses. The authors observed a significant suppression of microglial activation, leading to attenuation of the symptomatic phase and extended survival of *SOD1^G93A^* mice [141]. Notably, given their limited infiltration into the CNS, it is intriguing that peripheral macrophages have such a profound impact on CNS microglia activation. To gain further insight into the functional role of peripheral macrophages and monocytes on CNS cells, multicellular co-cultures of iPSC-derived cells could be a valuable tool as they simulate the complex interactions occurring between peripheral and CNS cells in vivo.

### 4.2 T cell

In addition to myeloid cells, early changes in CD4^+^ T cells isolated from peripheral blood samples of ALS patients have been found to be significantly correlated with disease progression, as measured by the decline in revised ALS functional rating scale (ALSFRS-R) [151]. Infiltrated CD4^+^ T cells have been shown to provide neuroprotection and prolong the survival of mutant *SOD1* mice by modulating glial cell phenotypes [152, 153]. Specifically, Treg cells are increased in number during the early stages, inducing neuroprotective M2 microglia and suppressing effector T cells by enhancing the expression of IL-4, IL-10, and TGF-β [154]. However, as disease progression rapidly accelerates, pro-inflammatory Th1 cells and M1 microglia become dominant due to depleted or dysfunctional Treg cells [155]. The numbers of Treg cells and FoxP3 protein expression have been found to be inversely correlated with disease progression in ALS patients [20, 155-157] A preliminary phase I trial also observed a significant correlation between increased Treg suppressive function and slower disease progression [158].

In contrast to the neuroprotective CD4^+^T cells, studies have demonstrated that self-reactive CD8^+^ T cells, upon infiltration into the CNS, can accelerate neurodegeneration and decrease overall survival in *SOD1^G93A^* mutant mice, the cytotoxic effects of which require an MHCI-dependent interaction between motor neurons and CD8^+^ T cells [13]. The concept of a "dying backward" process has been proposed in ALS pathophysiology, where neurodegeneration initiates as distal axonopathy and progresses retrogradely, as the distal axons and neuromuscular junction are constantly exposed to circulating immune cells [159]. Research has also shown that the absence of microglial MHCI and CD8^+^ T cells in the PNS can have adverse effects on axon stability and hasten disease onset, as they actively participate in removing axon debris and enhancing axonal regrowth [160]. These dual roles of peripheral immunity in neurodegeneration offer a possible explanation for the lack of success of systemic immunoregulatory medications in treating ALS.

### 4.3 NK cell

An increase in NK cell number has been observed in the peripheral blood of ALS patients, and has been negatively associated with the risk of death in longitudinal cohort studies [151, 161]. Alterations in NK cell surface markers related to trafficking and cytotoxicity have been found to be associated with changes in the ALSFRS-R in a sex- and age-dependent manner, while depletion of NK cells has been shown to extend survival in female ALS mice [162]. Researchers have used mouse models and primary NK cells derived from human subjects to demonstrate that pharmaceutical inhibition of NK cell activation could potentially protect ALS neurons from NK cell-mediated cytotoxicity [163]. Mechanistically, under physiological conditions, the presentation of MHCI antigens on motor neurons prevents NK cells from engaging in signaling cascades that lead to lymphocytic toxicity [164]. However, astrocytes derived from ALS patients or mutant *SOD1* mice have been found to decrease the expression of MHCI by motor neuron, which acts as a trigger for NK cells to produce effector molecules that induce neurotoxicity [165]. In *SOD1* mouse models, the CCL2-dependent accumulation of NK cells in the motor cortex and spinal cord has been found to contribute to motor neuron degeneration by inducing M1 microglia polarization and impairing Treg cell recruitment [144]. Overall, the current evidence suggests a neurotoxic role for NK cells in ALS. However, further study is required to elucidate the specific mechanisms involved in NK cell-mediated neurotoxicity in ALS.

## 5 Therapeutic implication

The treatment of ALS has been challenging, with currently approved drugs like riluzole and edaravone providing only modest therapeutic benefits in terms of patient survival and progression rates. However, significant progress has been made in understanding the pathophysiology of ALS in the past decade, leading to the development of over 50 drug development programs that are currently underway [166]. These programs focus on developing novel therapeutic agents or reformulating existing drugs to target various aspects of ALS pathology. Some clinical trials in ALS have targeted motor neuron-intrinsic pathways that are implicated in the disease, such as impaired protein homeostasis, mitochondrial dysfunction, aberrant RNA metabolism, and dysregulated vesicle transport [4]. For example, the administration of Tofersen, which binds to the mRNA of mutant SOD1 and reduces the synthesis of the protein, has shown promise in slowing functional decline and achieving clinical stabilization in patients [167]. Another approach is the fixed-dose combination therapy of taurursodiol and sodium phenylbutyrate (AMX0035), which inhibits mitochondrial-associated apoptosis and histone deacetylase, and has demonstrated functional and survival benefits [168, 169]. Some latest reviews have provided comprehensive overviews of recent advances in ALS clinical trials and novel drug development [166, 170]. Since neuroinflammation is a prominent target category for ALS therapeutic strategies, in the following sections, the current landscape of ALS therapeutic approaches related to neuroinflammation will be described, highlighting the prospects and challenges of emerging genetic and cell-based therapies.

### 5.1 Genetic therapies

Genetic therapy in ALS can be categorized into three main approaches: silencing, editing and replacement [170]. Silencing methods, such as antisense oligonucleotides (ASOs) and RNA interference (RNAi), are used to silence genes with toxic GoF mutations; gene editing involves correcting genetic mutations using engineered nucleases like CRISPR- Cas9; gene replacement aims to deliver functional copies of LoF mutant genes [171]. For example, the intravenous infusion of an adeno-associated viral (AAV) vector containing full-length copies of survival motor neuron 1 (SMN1) has shown success in treating spinal muscular atrophy type 1 (SMA1), a disease characterized by motor neuron degeneration [171]. Given the similarities between SMA and ALS, the success of gene replacement strategies in SMA has inspired potential therapeutic options for ALS. Intracerebroventricular (ICV) injection of ASOs targeting GoF ALS mutants has already shown promise as a therapeutic strategy in genetic mouse models and ALS patients [167, 172-175].

Targeting neuroinflammation in ALS show therapeutic potential, with several genetic candidates exhibiting promise in vitro and in vivo. One such candidate is *TBK1*, which plays a critical role in regulating type I IFN immunity and NF-κB signaling. Haploinsufficiency of *TBK1* due to nonsense or frameshift mutations has been implicated in ALS pathology [176, 177]. Missense mutations in functional domains of *TBK1* can disrupt autophagy function by impairing its ability to phosphorylate IRF3, OPTN and SQSTM1 [178, 179]. Studies have demonstrated that ICV delivery of AAV-mediated *TBK1* gene replacement can significantly prolong survival and improve motor function in *SOD1^G93A^* mice [180]. Manipulation of endogenous OPTN expression, a negative regulator of NF-κB, through gene replacement therapy has shown potential benefits by increasing NF-κB activity and reducing neuronal death [181]. Similarly, gene replacement therapy targeting LoF mutations in *ANG* has also been suggested [182]. Therefore, genetic manipulation of genes implicated in neuroinflammation holds promise for providing beneficial effects in specific subsets of ALS patients.

While genetic therapy holds great potential for treating ALS, there are several challenges that need to be addressed. One major obstacle is effectively delivering the therapeutic agent to both neuronal and glial cells in the CNS, as ALS affects both cell types. Additionally, the therapeutic agent must efficiently pass the BBB to reach the target cells. AAV9 has shown promise as a vector due to its ability to cross the BBB and distribute throughout the CNS [183]. Another consideration is the potential adverse effects of targeting a single gene, as both LoF and GoF mechanisms are often associated with ALS pathogenesis. Modulating the expression of a single gene may have unintended consequences, emphasizing the necessity for a more comprehensive understanding of disease mechanisms before developing targeted genetic therapies. Future research should focus on identifying specific genes and pathways involved in disease pathogenesis to refine genetic therapies, maximizing therapeutic benefits while minimizing risks.

### 5.2 Cell-based therapies

Despite the growing body of evidence from both clinical and preclinical studies supporting the active role of neuroinflammation in ALS pathogenesis, immuno-suppression interventions have shown limited efficacy in terms of disease progression and patient survival [184]. One possible explanation is that the administration of untargeted immunomodulatory drugs fails to effectively modulate the relative proportion of proinflammatory effector T cells and anti-inflammatory Treg cells. Consequently, cell-based strategies have emerged as a promising avenue for the development of more effective therapeutic interventions. The rationale behind cell-based therapies lies in their potential to modulate neuroinflammatory pathways or secrete neuroprotective factors by selectively targeting specific cell types.

Studies assessing cell-based therapies have revealed promising therapeutic potential for ALS patients. For example, intravenous infusions of ex vivo expanded autologous Treg cells, accompanied by IL-2 injections during both early and later stages of the disease, have been deemed well-tolerated in patients and correlated with a slower decline in the ALSFRS-R [158]. Currently, the therapeutic effects of infusing autologous hybrid T cells are being evaluated in a larger ongoing phase 1/2 cohort study (NCT04220190). In addition, intrathecal injection of functional astrocytes (AstroRx®) in ALS patients has been established as safe and beneficial in an open-label, phase 1/2a clinical trial [185]. Transplantation of neural progenitor cells transduced with glial cell line-derived neurotrophic factor (GDNF) has also met the safety endpoint in a phase 3 clinical trial, where they differentiated into astrocytes and provided trophic support to spinal cord neurons [186]. Similarly, transplantation of autologous mesenchymal stem cells (MSCs) induced with neurotrophic factors (NurOwn) has also demonstrated promising therapeutic potential in a phase 2 randomized controlled trial [187]. Furthermore, a phase 3 clinical trial has supported the safety and efficacy of intrathecal administration of autologous MSC, showing a statistically significant reduction of neuroinflammation and neurodegeneration biomarkers in ALS CSF [188]. Ongoing studies are evaluating the long-term safety and efficacy of MSC injection in a double-blind randomized phase 3 clinical trial (NCT04745299).

Additionally, drugs targeting immune cells or inflammatory pathways have demonstrated promising effects in the treatment of ALS. For example, in a phase 2a randomized trial, low-dose recombinant human IL-2 (Aldesleukin) significantly increased the percentage of Treg cells in CD4^+^ T cells of ALS patients [189]. RNS60 has demonstrated therapeutic efficacy in *SOD1^G93A^* mice by elevating peripheral Treg cell numbers and activating pro-survival pathways in neurons [190]. Its beneficial effects on ALS patients have recently been confirmed in a phase 2 randomized trial [191]. Masitinib, a selective tyrosine kinase inhibitor, downregulates proinflammatory cytokines and modulate neuroinflammation by targeting macrophages, mast cells, and microglia [192]. Oral Masitinib significantly delays the decline of ALSFRS-R and prolongs survival in ALS patients [193, 194]. The safety and efficacy of Ibudilast, which inhibits neuroinflammation by attenuating macrophage migration and glial cell activation, are currently being assessed in an ongoing phase 2b/3 clinical trial [195]. Fasudil, a rho kinase inhibitor, has been shown to modulate microglia activation and prolong survival in *SOD1^G93A^* mice by reducing the release of proinflammatory cytokines and chemokines [196]. Its safety and efficacy are currently being evaluated in a phase 2 clinical trial (NCT05218668).

## 6 Conclusions and outlook

There is a growing body of evidence indicating the critical role of neuroinflammation in ALS pathogenesis, and recent years have seen notable progress in understanding the disease. Genetic investigations have identified several susceptibility genes associated with immune function in ALS, providing strong evidence for the involvement of inflammation in the disease process. However, the inflammatory response in ALS is complex and dynamic, encompassing various molecular and cellular pathways. The interplay between neuroinflammation and neurodegeneration establishes a detrimental cycle, where inflammation-induced neuronal damage further amplifies the inflammatory response. The roles of different immune cells, such as microglia, astrocytes, macrophages, T cells, and NK cells, in both the brain and peripheral tissues, have been well-established in the neuroinflammatory response in ALS, and extensive research has been conducted to understand their contributions to disease progression and pathology. Consequently, targeting inflammation in ALS holds considerable therapeutic potential, leading to the exploration of various approaches, including immune cell-specific interventions, modulation of neuroinflammatory signaling pathways, and genetic therapy. Some of these approaches have shown promising results in preclinical studies, and clinical trials are currently underway to evaluate their safety and efficacy.

Despite the advances in understanding the role of neuroinflammation in ALS, there is still much to be investigated. The activation of glial cells has been viewed as either homeostatic at early disease stages or neurotoxic as the disease progresses, although the exact triggers for the phenotypic transition remain unclear. Unraveling the molecular changes in microglia and astrocytes at different stages of the disease could help determine their contributions to ALS progression. Furthermore, understanding the interaction between genetic and environmental factors and their effect on the neuroinflammatory response in ALS requires further clarification. Novel technologies like single-cell RNA sequencing and multiomics platforms hold promise for better comprehending the molecular and cellular mechanisms of neuroinflammation in ALS. Ultimately, it is crucial to develop more effective therapeutic strategies that specifically target neuroinflammation in ALS. While some promising approaches have emerged and undergone testing, gaining a better understanding of ALS pathogenesis and identifying new therapeutic targets are still essential. A personalized approach to therapy, considering the patient's genetic and immune profile, may also prove beneficial.

In conclusion, neuroinflammation is a critical factor in the pathogenesis of ALS, and further studies are needed to elucidate the underlying mechanisms and develop effective treatments. The development of targeted therapies that modulate the inflammatory response in ALS has the potential to slow or halt disease progression, thereby improving the quality of life for ALS patients.

## References

[1] Ryan M, Heverin M, McLaughlin RL, Hardiman O (2019). Lifetime Risk and Heritability of Amyotrophic Lateral Sclerosis. JAMA Neurol, 1-8. 10.1001/jamaneurol.2019.2044 PMC6646974 31329211

[2] Feldman EL, Goutman SA, Petri S, Mazzini L, Savelieff MG, Shaw PJ, et al. (2022). Amyotrophic lateral sclerosis. Lancet, 400:1363-1380. 36116464 10.1016/S0140-6736(22)01272-7 PMC10089700

[3] van Es MA, Hardiman O, Chio A, Al-Chalabi A, Pasterkamp RJ, Veldink JH, et al. (2017). Amyotrophic lateral sclerosis. Lancet, 390:2084-2098. 28552366 10.1016/S0140-6736(17)31287-4

[4] Hardiman O, Al-Chalabi A, Chio A, Corr EM, Logroscino G, Robberecht W, et al. (2017). Amyotrophic lateral sclerosis. Nat Rev Dis Prim, 3:17071. 28980624 10.1038/nrdp.2017.71

[5] Brown RH, Al-Chalabi A (2017). Amyotrophic Lateral Sclerosis. N Engl J Med, 377:162-172. 28700839 10.1056/NEJMra1603471

[6] Swinnen B, Robberecht W (2014). The phenotypic variability of amyotrophic lateral sclerosis. Nat Rev Neurol, 10:661-670. 25311585 10.1038/nrneurol.2014.184

[7] Goutman SA, Hardiman O, Al-Chalabi A, Chió A, Savelieff MG, Kiernan MC, et al. (2022). Recent advances in the diagnosis and prognosis of amyotrophic lateral sclerosis. Lancet Neurol, 21:480-493. 35334233 10.1016/S1474-4422(21)00465-8 PMC9513753

[8] Crockford C, Newton J, Lonergan K, Chiwera T, Booth T, Chandran S, et al. (2018). ALS-specific cognitive and behavior changes associated with advancing disease stage in ALS. Neurology, 91:E1370-E1380. 30209236 10.1212/WNL.0000000000006317 PMC6177274

[9] Goutman SA, Hardiman O, Al-Chalabi A, Chió A, Savelieff MG, Kiernan MC, et al. (2022). Emerging insights into the complex genetics and pathophysiology of amyotrophic lateral sclerosis. Lancet Neurol, 21:465-479. 35334234 10.1016/S1474-4422(21)00414-2 PMC9513754

[10] Chia R, Chiò A, Traynor BJ (2018). Novel genes associated with amyotrophic lateral sclerosis: diagnostic and clinical implications. Lancet Neurol, 17:94-102. 29154141 10.1016/S1474-4422(17)30401-5 PMC5901717

[11] Beers DR, Appel SH (2019). Immune dysregulation in amyotrophic lateral sclerosis: mechanisms and emerging therapies. Lancet Neurol, 18:211-220. 30663610 10.1016/S1474-4422(18)30394-6

[12] McCauley ME, Baloh RH (2019). Inflammation in ALS/FTD pathogenesis. Acta Neuropathol, 137:715-730. 30465257 10.1007/s00401-018-1933-9 PMC6482122

[13] Coque E, Salsac C, Espinosa-Carrasco G, Varga B, Degauque N, Cadoux M, et al. (2019). Cytotoxic CD8 + T lymphocytes expressing ALS-causing SOD1 mutant selectively trigger death of spinal motoneurons. Proc Natl Acad Sci U S A, 116:2312-2317. 30674678 10.1073/pnas.1815961116 PMC6369778

[14] Brettschneider J, Toledo JB, van Deerlin VM, Elman L, McCluskey L, Lee VMY, et al. (2012). Microglial activation correlates with disease progression and upper motor neuron clinical symptoms in amyotrophic lateral sclerosis. PLoS One, 7:13-15. 10.1371/journal.pone.0039216 PMC3375234 22720079

[15] Garbuzova-Davis S, Sanberg PR (2014). Blood-CNS barrier impairment in ALS patients versus an animal model. Front Cell Neurosci, 8:1-9. 24550780 10.3389/fncel.2014.00021 PMC3910123

[16] Engelhardt JI, Tajti J, Appel SH (1993). Lymphocytic Infiltrates in the Spinal Cord in Amyotrophic Lateral Sclerosis. Arch Neurol, 50:30-36. 8093428 10.1001/archneur.1993.00540010026013

[17] Kawamata T, Akiyama H, Yamada T, McGeer PL (1992). Immunologic reactions in amyotrophic lateral sclerosis brain and spinal cord tissue. Am J Pathol, 140:691-707. 1347673 PMC1886170

[18] Henkel JS, Engelhardt JI, Siklós L, Simpson EP, Kim SH, Pan T, et al. (2004). Presence of Dendritic Cells, MCP-1, and Activated Microglia/Macrophages in Amyotrophic Lateral Sclerosis Spinal Cord Tissue. Ann Neurol, 55:221-235. 14755726 10.1002/ana.10805

[19] Zhang R, Gascon R, Miller RG, Gelinas DF, Mass J, Hadlock K, et al. (2005). Evidence for systemic immune system alterations in sporadic amyotrophic lateral sclerosis (sALS). J Neuroimmunol, 159:215-224. 15652422 10.1016/j.jneuroim.2004.10.009

[20] Beers DR, Zhao W, Wang J, Zhang X, Wen S, Neal D, et al. (2017). ALS patients’ regulatory T lymphocytes are dysfunctional, and correlate with disease progression rate and severity. JCI Insight, 2:1-14. 10.1172/jci.insight.89530 PMC5333967 28289705

[21] Lu C-H, Allen K, Oei F, Leoni E, Kuhle J, Tree T, et al. (2016). Systemic inflammatory response and neuromuscular involvement in amyotrophic lateral sclerosis. Neurol - Neuroimmunol Neuroinflammation, 3:e244. 10.1212/NXI.0000000000000244 PMC4897985 27308305

[22] Staats KA, Borchelt DR, Tansey MG, Wymer J (2022). Blood-based biomarkers of inflammation in amyotrophic lateral sclerosis. Mol Neurodegener, 17:1-19. 35073950 10.1186/s13024-022-00515-1 PMC8785449

[23] Zhao W, Beers DR, Hooten KG, Sieglaff DH, Zhang A, Kalyana-Sundaram S, et al. (2017). Characterization of Gene Expression Phenotype in Amyotrophic Lateral Sclerosis Monocytes. JAMA Neurol, 74:677. 28437540 10.1001/jamaneurol.2017.0357 PMC5822209

[24] Rusconi M, Gerardi F, Santus W, Lizio A, Sansone VA, Lunetta C, et al. (2017). Inflammatory role of dendritic cells in Amyotrophic Lateral Sclerosis revealed by an analysis of patients’ peripheral blood. Sci Rep, 7:1-9. 28798369 10.1038/s41598-017-08233-1 PMC5552769

[25] Swarup V, Phaneuf D, Dupré N, Petri S, Strong M, Kriz J, et al. (2011). Deregulation of TDP-43 in amyotrophic lateral sclerosis triggers nuclear factor κB-mediated pathogenic pathways. J Exp Med, 208:2429-2447. 22084410 10.1084/jem.20111313 PMC3256969

[26] Wang R, Yang B, Zhang D (2011). Activation of interferon signaling pathways in spinal cord astrocytes from an ALS mouse model. Glia, 59:946-958. 21446050 10.1002/glia.21167 PMC3077460

[27] Maruyama H, Morino H, Ito H, Izumi Y, Kato H, Watanabe Y, et al. (2010). Mutations of optineurin in amyotrophic lateral sclerosis. Nature, 465:223-226. 20428114 10.1038/nature08971

[28] Lee SH, Kim KW, Min KM, Kim KW, Chang SI, Kim JC (2014). Angiogenin reduces immune inflammation via inhibition of tank-binding kinase 1 expression in human corneal fibroblast cells. Mediators Inflamm. doi: 10.1155/2014/861435. PMC4016892 24860242

[29] Fecto F, Yan J, Vemula SP, Liu E, Yang Y, Chen W, et al. (2011). SQSTM1 mutations in familial and sporadic amyotrophic lateral sclerosis. Arch Neurol, 68:1440-6. 22084127 10.1001/archneurol.2011.250

[30] Johnson JO, Mandrioli J, Benatar M, Abramzon Y, Van Deerlin VM, Trojanowski JQ, et al. (2010). Exome Sequencing Reveals VCP Mutations as a Cause of Familial ALS. Neuron, 68:857-864. 21145000 10.1016/j.neuron.2010.11.036 PMC3032425

[31] Gallagher ER, Holzbaur ELF (2023). The selective autophagy adaptor p62/SQSTM1 forms phase condensates regulated by HSP27 that facilitate the clearance of damaged lysosomes via lysophagy. Cell Rep, 42:112037. 36701233 10.1016/j.celrep.2023.112037 PMC10366342

[32] Cirulli ET, Lasseigne BN, Petrovski S, Sapp PC, Dion PA, Leblond CS, et al. (2015). Exome sequencing in amyotrophic lateral sclerosis identifies risk genes and pathways. Science (80- ), 347:1436-1441. 10.1126/science.aaa3650 PMC4437632 25700176

[33] Szegö EM, Malz L, Bernhardt N, Rösen-Wolff A, Falkenburger BH, Luksch H (2022). Constitutively active STING causes neuroinflammation and degeneration of dopaminergic neurons in mice. Elife, 11:1-30. 10.7554/eLife.81943 PMC9767458 36314770

[34] Yu CH, Davidson S, Harapas CR, Hilton JB, Mlodzianoski MJ, Laohamonthonkul P, et al. (2020). TDP-43 Triggers Mitochondrial DNA Release via mPTP to Activate cGAS/STING in ALS. Cell, 183:636-649.e18. 33031745 10.1016/j.cell.2020.09.020 PMC7599077

[35] Van Damme P, Robberecht W (2021). STING-Induced Inflammation — A Novel Therapeutic Target in ALS? N Engl J Med, 384:765-767. 33626258 10.1056/NEJMcibr2031048

[36] Neumann M, Sampathu DM, Kwong LK, Truax AC, Micsenyi MC, Chou TT, et al. (2006). Ubiquitinated TDP-43 in frontotemporal lobar degeneration and amyotrophic lateral sclerosis. Science (80- ), 314:130-133. 10.1126/science.1134108 17023659

[37] Xie M, Liu YU, Zhao S, Zhang L, Bosco DB, Pang YP, et al. (2022). TREM2 interacts with TDP-43 and mediates microglial neuroprotection against TDP-43-related neurodegeneration. Nat Neurosci, 25:26-38. 34916658 10.1038/s41593-021-00975-6 PMC8741737

[38] Wang W, Li L, Lin WL, Dickson DW, Petrucelli L, Zhang T, et al. (2013). The ALS disease-associated mutant TDP-43 impairs mitochondrial dynamics and function in motor neurons. Hum Mol Genet, 22:4706-4719. 23827948 10.1093/hmg/ddt319 PMC3820133

[39] Zhao B, Xu P, Rowlett CM, Jing T, Shinde O, Lei Y, et al. (2020). The molecular basis of tight nuclear tethering and inactivation of cGAS. Nature, 587:673-677. 32911481 10.1038/s41586-020-2749-z PMC7704945

[40] Ablasser A, Chen ZJ (2019). CGAS in action: Expanding roles in immunity and inflammation. Science (80- ). doi: 10.1126/science.aat8657. 30846571

[41] Zhang C, Shang G, Gui X, Zhang X, Chen Bai X, Chen ZJ (2019). Structural basis of STING binding with and phosphorylation by TBK1. Nature, 567:394-398. 30842653 10.1038/s41586-019-1000-2 PMC6862768

[42] McCauley ME, O’Rourke JG, Yáñez A, Markman JL, Ho R, Wang X, et al. (2020). C9orf72 in myeloid cells suppresses STING-induced inflammation. Nature, 585:96-101. 32814898 10.1038/s41586-020-2625-x PMC7484469

[43] Wang L, Wen M, Cao X (2019). Nuclear hnRNPA2B1 initiates and amplifies the innate immune response to DNA viruses. Science (80- ), 365:1-18. 10.1126/science.aav0758 31320558

[44] Xiao S, Cao S, Huang Q, Xia L, Deng M, Yang M, et al. (2019). The RNA N6-methyladenosine modification landscape of human fetal tissues. Nat Cell Biol, 21:651-661. 31036937 10.1038/s41556-019-0315-4

[45] Tank EM, Figueroa-Romero C, Hinder LM, Bedi K, Archbold HC, Li X, et al. (2018). Abnormal RNA stability in amyotrophic lateral sclerosis. Nat Commun. doi: 10.1038/s41467-018-05049-z. PMC6054632 30030424

[46] Zaccara S, Ries RJ, Jaffrey SR (2019). Reading, writing and erasing mRNA methylation. Nat Rev Mol Cell Biol, 20:608-624. 31520073 10.1038/s41580-019-0168-5

[47] Huang H, Weng H, Chen J (2020). The Biogenesis and Precise Control of RNA m6A Methylation. Trends Genet, 36:44-52. 31810533 10.1016/j.tig.2019.10.011 PMC6925345

[48] Xiong X, Hou L, Park YP, Molinie B, Ardlie KG, Aguet F, et al. (2021). Genetic drivers of m6A methylation in human brain, lung, heart and muscle. Nat Genet, 53:1156-1165. 34211177 10.1038/s41588-021-00890-3 PMC9112289

[49] McMillan M, Gomez N, Hsieh C, Bekier M, Li X, Miguez R, et al. (2023). RNA methylation influences TDP43 binding and disease pathogenesis in models of amyotrophic lateral sclerosis and frontotemporal dementia. Mol Cell, 2022.04.03.486880. 10.1016/j.molcel.2022.12.019 PMC9899051 36634675

[50] Pineda, Sebastian S, Lee Hyeseung, Fitzwalter Brent E, Mohammadi Shahin, Pregent Luc J, Mahammad E Gardashli JM et al. (2021). Single-cell profiling of the human primary motor cortex in ALS and FTLD. bioRvix. doi: 10.1101/2021.07.07.451374.

[51] Winkler R, Gillis E, Lasman L, Safra M, Geula S, Soyris C, et al. (2019). m 6 A modification controls the innate immune response to infection by targeting type I interferons. Nat Immunol, 20:173-182. 30559377 10.1038/s41590-018-0275-z

[52] Xiao W, Adhikari S, Dahal U, Chen YS, Hao YJ, Sun BF, et al. (2016). Nuclear m6A Reader YTHDC1 Regulates mRNA Splicing. Mol Cell, 61:507-519. 26876937 10.1016/j.molcel.2016.01.012

[53] Edens BM, Vissers C, Su J, Arumugam S, Xu Z, Shi H, et al. (2019). FMRP Modulates Neural Differentiation through m6A-Dependent mRNA Nuclear Export. Cell Rep, 28:845-854.e5. 31340148 10.1016/j.celrep.2019.06.072 PMC6687293

[54] Roundtree IA, Luo GZ, Zhang Z, Wang X, Zhou T, Cui Y, et al. (2017). YTHDC1 mediates nuclear export of N6-methyladenosine methylated mRNAs. Elife, 6:1-28. 10.7554/eLife.31311 PMC5648532 28984244

[55] Choi J, Ieong KW, Demirci H, Chen J, Petrov A, Prabhakar A, et al. (2016). N6-methyladenosine in mRNA disrupts tRNA selection and translation-elongation dynamics. Nat Struct Mol Biol, 23:110-115. 26751643 10.1038/nsmb.3148 PMC4826618

[56] Meyer KD, Patil DP, Zhou J, Zinoviev A, Skabkin MA, Elemento O, et al. (2015). 5′ UTR m6A Promotes Cap-Independent Translation. Cell, 163:999-1010. 26593424 10.1016/j.cell.2015.10.012 PMC4695625

[57] Wang Y, Li Y, Toth JI, Petroski MD, Zhang Z, Zhao JC (2014). N6 -methyladenosine modification destabilizes developmental regulators in embryonic stem cells. Nat Cell Biol, 16:191-198. 24394384 10.1038/ncb2902 PMC4640932

[58] Shi H, Wang X, Lu Z, Zhao BS, Ma H, Hsu PJ, et al. (2017). YTHDF3 facilitates translation and decay of N 6-methyladenosine-modified RNA. Cell Res, 27:315-328. 28106072 10.1038/cr.2017.15 PMC5339834

[59] Wang X, Zhao BS, Roundtree IA, Lu Z, Han D, Ma H, et al. (2015). N6-methyladenosine modulates messenger RNA translation efficiency. Cell, 161:1388-1399. 26046440 10.1016/j.cell.2015.05.014 PMC4825696

[60] Zhao BS, Roundtree IA, He C (2016). Post-transcriptional gene regulation by mRNA modifications. Nat Rev Mol Cell Biol, 18:31-42. 27808276 10.1038/nrm.2016.132 PMC5167638

[61] Boulias K, Greer EL (2023). Biological roles of adenine methylation in RNA. Nat Rev Genet, 24:143-160. 36261710 10.1038/s41576-022-00534-0 PMC9974562

[62] Roundtree IA, Evans ME, Pan T, He C (2017). Dynamic RNA Modifications in Gene Expression Regulation. Cell, 169:1187-1200. 28622506 10.1016/j.cell.2017.05.045 PMC5657247

[63] Cui L, Ma R, Cai J, Guo C, Chen Z, Yao L, et al. (2022). RNA modifications: importance in immune cell biology and related diseases. Signal Transduct Target Ther. doi: 10.1038/s41392-022-01175-9. PMC9499983 36138023

[64] Zhu X, Tang HJ, Yang M, Yin K (2023). N6-methyladenosine in macrophage function: a novel target for metabolic diseases. Trends Endocrinol Metab, 34:66-84. 36586778 10.1016/j.tem.2022.12.006

[65] Tong J, Wang X, Liu Y, Ren X, Wang A, Chen Z, et al. (2021). Pooled CRISPR screening identifies m6A as a positive regulator of macrophage activation. Sci Adv. doi: 10.1126/sciadv.abd4742. PMC8081357 33910903

[66] Qin Y, Li B, Arumugam S, Lu Q, Mankash SM, Li J, et al. (2021). m6A mRNA methylation-directed myeloid cell activation controls progression of NAFLD and obesity. Cell Rep, 37:109968. 34758326 10.1016/j.celrep.2021.109968 PMC8667589

[67] Zheng Q, Hou J, Zhou Y, Li Z, Cao X (2017). The RNA helicase DDX46 inhibits innate immunity by entrapping m 6 A-demethylated antiviral transcripts in the nucleus. Nat Immunol, 18:1094-1103. 28846086 10.1038/ni.3830

[68] Rubio RM, Depledge DP, Bianco C, Thompson L, Mohr I (2018). RNA m 6 A modification enzymes shape innate responses to DNA by regulating interferon β. Genes Dev, 32:1472-1484. 30463905 10.1101/gad.319475.118 PMC6295168

[69] Du J, Liao W, Liu W, Deb DK, He L, Hsu PJ, et al. (2020). N6-Adenosine Methylation of Socs1 mRNA Is Required to Sustain the Negative Feedback Control of Macrophage Activation. Dev Cell, 55:737-753.e7. 33220174 10.1016/j.devcel.2020.10.023 PMC7755741

[70] Yao Y, Yang Y, Guo W, Xu L, You M, Zhang YC, et al. (2021). METTL3-dependent m6A modification programs T follicular helper cell differentiation. Nat Commun, 12:1-16. 33637761 10.1038/s41467-021-21594-6 PMC7910450

[71] Li H-B, Tong J, Zhu S, Batista PJ, Duffy EE, Zhao J, et al. (2017). m6A mRNA methylation controls T cell homeostasis by targeting the IL-7/STAT5/SOCS pathways. Nature, 548:338-342. 28792938 10.1038/nature23450 PMC5729908

[72] Tong J, Cao G, Zhang T, Sefik E, Vesely MCA, Broughton JP, et al. (2018). M6 A mRNA methylation sustains Treg suppressive functions. Cell Res, 28:253-256. 29303144 10.1038/cr.2018.7 PMC5799823

[73] Lu TX, Zheng Z, Zhang L, Sun HL, Bissonnette M, Huang H, et al. (2020). A New Model of Spontaneous Colitis in Mice Induced by Deletion of an RNA m6A Methyltransferase Component METTL14 in T Cells. Cmgh, 10:747-761. 32634481 10.1016/j.jcmgh.2020.07.001 PMC7498954

[74] Shulman Z, Stern-Ginossar N (2020). The RNA modification N 6-methyladenosine as a novel regulator of the immune system. Nat Immunol, 21:501-512. 32284591 10.1038/s41590-020-0650-4

[75] Zhou J, Zhang X, Hu J, Qu R, Yu Z, Xu H, et al. (2021). M6A demethylase ALKBH5 controls CD4+T cell pathogenicity and promotes autoimmunity. Sci Adv, 7:1-14. 10.1126/sciadv.abg0470 PMC8208713 34134995

[76] DeJesus-Hernandez M, Mackenzie IR, Boeve BF, Boxer AL, Baker M, Rutherford NJ, et al. (2011). Expanded GGGGCC Hexanucleotide Repeat in Noncoding Region of C9ORF72 Causes Chromosome 9p-Linked FTD and ALS. Neuron, 72:245-256. 21944778 10.1016/j.neuron.2011.09.011 PMC3202986

[77] O’Rourke JG, Bogdanik L, Yáñez A, Lall D, Wolf AJ, Muhammad AKMG, et al. (2016). C9orf72 is required for proper macrophage and microglial function in mice. Science (80- ), 351:1324-1329. 10.1126/science.aaf1064 PMC5120541 26989253

[78] Nataf S, Pays L (2015). Gene co-expression analysis unravels a link between C9orf72 and RNA metabolism in myeloid cells. Acta Neuropathol Commun, 3:64. 26472214 10.1186/s40478-015-0242-y PMC4608290

[79] Rizzu P, Blauwendraat C, Heetveld S, Lynes EM, Castillo-Lizardo M, Dhingra A, et al. (2016). C9orf72 is differentially expressed in the central nervous system and myeloid cells and consistently reduced in C9orf72, MAPT and GRN mutation carriers. Acta Neuropathol Commun, 4:37. 27079381 10.1186/s40478-016-0306-7 PMC4832459

[80] Burberry A, Suzuki N, Wang JY, Moccia R, Mordes DA, Stewart MH, et al. (2016). Loss-of-function mutations in the C9ORF72 mouse ortholog cause fatal autoimmune disease. Sci Transl Med. doi: 10.1126/scitranslmed.aaf6038. PMC5024536 27412785

[81] Lall D, Baloh RH (2017). Microglia and C9orf72 in neuroinflammation and ALS and frontotemporal dementia. J Clin Invest, 127:3250-3258. 28737506 10.1172/JCI90607 PMC5669558

[82] Sudria-Lopez E, Koppers M, de Wit M, van der Meer C, Westeneng HJ, Zundel CAC, et al. (2016). Full ablation of C9orf72 in mice causes immune system-related pathology and neoplastic events but no motor neuron defects. Acta Neuropathol, 132:145-147. 27206760 10.1007/s00401-016-1581-x PMC4911370

[83] La Spada AR, Taylor JP (2010). Repeat expansion disease: progress and puzzles in disease pathogenesis. Nat Rev Genet, 11:247-258. 20177426 10.1038/nrg2748 PMC4704680

[84] Shao W, Todd TW, Wu Y, Jones CY, Tong J, Jansen-West K, et al. (2022). Two FTD-ALS genes converge on the endosomal pathway to induce TDP-43 pathology and degeneration. Science (80- ), 378:94-99. 10.1126/science.abq7860 PMC9942492 36201573

[85] Cook CN, Wu Y, Odeh HM, Gendron TF, Jansen-West K, del Rosso G, et al. (2020). C9orf72 poly(GR) aggregation induces TDP-43 proteinopathy. Sci Transl Med, 12:498-503. 10.1126/scitranslmed.abb3774 PMC7989020 32878979

[86] Khosravi B, Hartmann H, May S, Möhl C, Ederle H, Michaelsen M, et al. (2017). Cytoplasmic poly-GA aggregates impair nuclear import of TDP-43 in C9orf72 ALS/FTLD. Hum Mol Genet, 26:790-800. 28040728 10.1093/hmg/ddw432 PMC5409121

[87] Shu X, Wei C, Tu W, Zhong K, Qi S, Wang A, et al. (2023). Report Negative regulation of TREM2-mediated C9orf72 poly-GA clearance by the NLRP3 inflammasome ll ll Negative regulation of TREM2-mediated C9orf72 poly-GA clearance by the NLRP3 inflammasome. CellReports, 42:112133. 10.1016/j.celrep.2023.112133 36800288

[88] Rosen D (1993). Mutations in Cu/Zn superoxide dismutase gene are associated with familial amyotrophic lateral sclerosis. Nature, 364:362-362. 8332197 10.1038/364362c0

[89] Pramatarova A, Laganière J, Roussel J, Brisebois K, Rouleau GA (2001). Neuron-specific expression of mutant superoxide dismutase 1 in transgenic mice does not lead to motor impairment. J Neurosci, 21:3369-3374. 11331366 10.1523/JNEUROSCI.21-10-03369.2001 PMC6762496

[90] Boillée S, Yamanaka K, Lobsiger CS, Copeland NG, Jenkins NA, Kassiotis G, et al. (2006). Onset and progression in inherited ALS determined by motor neurons and microglia. Science (80- ), 312:1389-1392. 10.1126/science.1123511 16741123

[91] Clement AM, Nguyen MD, Roberts EA, Garcia ML, Boillée S, Rule M, et al. (2003). Wild-type nonneuronal cells extend survival of SOD1 mutant motor neurons in ALS mice. Science (80- ), 302:113-117. 10.1126/science.1086071 14526083

[92] Meissner F, Molawi K, Zychlinsky A (2010). Mutant superoxide dismutase 1-induced IL-1β accelerates ALS pathogenesis. Proc Natl Acad Sci U S A, 107:13046-13050. 20616033 10.1073/pnas.1002396107 PMC2919927

[93] Weydt P, Yuen EC, Ransom BR, Möller T (2004). Increased cytotoxic potential of microglia from ALS-transgenic mice. Glia, 48:179-182. 15378658 10.1002/glia.20062

[94] Xiao Q, Zhao W, Beers DR, Yen AA, Xie W, Henkel JS, et al. (2007). Mutant SOD1G93A microglia are more neurotoxic relative to wild-type microglia. J Neurochem, 102:2008-2019. 17555556 10.1111/j.1471-4159.2007.04677.x

[95] Frakes AE, Ferraiuolo L, Haidet-Phillips AM, Schmelzer L, Braun L, Miranda CJ, et al. (2014). Microglia Induce Motor Neuron Death via the Classical NF-κB Pathway in Amyotrophic Lateral Sclerosis. Neuron, 81:1009-1023. 24607225 10.1016/j.neuron.2014.01.013 PMC3978641

[96] Gerbino V, Kaunga E, Ye J, Canzio D, O’Keeffe S, Rudnick ND, et al. (2020). The Loss of TBK1 Kinase Activity in Motor Neurons or in All Cell Types Differentially Impacts ALS Disease Progression in SOD1 Mice. Neuron, 106:789-805.e5. 32220666 10.1016/j.neuron.2020.03.005

[97] Jara JH, Gautam M, Kocak N, Xie EF, Mao Q, Bigio EH, et al. (2019). MCP1-CCR2 and neuroinflammation in the ALS motor cortex with TDP-43 pathology. J Neuroinflammation, 16:196. 31666087 10.1186/s12974-019-1589-y PMC6822373

[98] Humphrey J, Venkatesh S, Hasan R, Herb JT, de Paiva Lopes K, Küçükali F, et al. (2023). Integrative transcriptomic analysis of the amyotrophic lateral sclerosis spinal cord implicates glial activation and suggests new risk genes. Nat Neurosci, 26:150-162. 36482247 10.1038/s41593-022-01205-3

[99] Pelvig DP, Pakkenberg H, Stark AK, Pakkenberg B (2008). Neocortical glial cell numbers in human brains. Neurobiol Aging, 29:1754-1762. 17544173 10.1016/j.neurobiolaging.2007.04.013

[100] Li Q, Barres BA (2018). Microglia and macrophages in brain homeostasis and disease. Nat Rev Immunol, 18:225-242. 29151590 10.1038/nri.2017.125

[101] Butovsky O, Weiner HL (2018). Microglial signatures and their role in health and disease. Nat Rev Neurosci, 19:622-635. 30206328 10.1038/s41583-018-0057-5 PMC7255106

[102] Brettschneider J, Libon DJ, Toledo JB, Xie SX, McCluskey L, Elman L, et al. (2012). Microglial activation and TDP-43 pathology correlate with executive dysfunction in amyotrophic lateral sclerosis. Acta Neuropathol, 123:395-407. 22210083 10.1007/s00401-011-0932-x PMC3595560

[103] Tondo G, Iaccarino L, Cerami C, Vanoli GE, Presotto L, Masiello V, et al. (2020). 11C-PK11195 PET-based molecular study of microglia activation in SOD1 amyotrophic lateral sclerosis. Ann Clin Transl Neurol, 7:1513-1523. 32762033 10.1002/acn3.51112 PMC7480909

[104] Alshikho MJ, Zürcher NR, Loggia ML, Cernasov P, Reynolds B, Pijanowski O, et al. (2018). Integrated magnetic resonance imaging and [ 11 C]-PBR28 positron emission tomographic imaging in amyotrophic lateral sclerosis. Ann Neurol, 83:1186-1197. 29740862 10.1002/ana.25251 PMC6105567

[105] Ransohoff RM (2016). A polarizing question: Do M1 and M2 microglia exist. Nat Neurosci, 19:987-991. 27459405 10.1038/nn.4338

[106] Liao B, Zhao W, Beers DR, Henkel JS, Appel SH (2012). Transformation from a neuroprotective to a neurotoxic microglial phenotype in a mouse model of ALS. Exp Neurol, 237:147-152. 22735487 10.1016/j.expneurol.2012.06.011 PMC4126417

[107] Gravel M, Béland LC, Soucy G, Abdelhamid E, Rahimian R, Gravel C, et al. (2016). Il-10 controls early microglial phenotypes and disease onset in ALS caused by misfolded superoxide dismutase 1. J Neurosci, 36:1031-1048. 26791230 10.1523/JNEUROSCI.0854-15.2016 PMC6601999

[108] Spiller KJ, Restrepo CR, Khan T, Dominique MA, Fang TC, Canter RG, et al. (2018). Microglia-mediated recovery from ALS-relevant motor neuron degeneration in a mouse model of TDP-43 proteinopathy. Nat Neurosci, 21:329-340. 29463850 10.1038/s41593-018-0083-7 PMC5857237

[109] Svahn AJ, Don EK, Badrock AP, Cole NJ, Graeber MB, Yerbury JJ, et al. (2018). Nucleo-cytoplasmic transport of TDP-43 studied in real time: impaired microglia function leads to axonal spreading of TDP-43 in degenerating motor neurons. Acta Neuropathol, 136:445-459. 29943193 10.1007/s00401-018-1875-2 PMC6096729

[110] Chiu IM, Morimoto ETA, Goodarzi H, Liao JT, O’Keeffe S, Phatnani HP, et al. (2013). A neurodegeneration-specific gene-expression signature of acutely isolated microglia from an amyotrophic lateral sclerosis mouse model. Cell Rep, 4:385-401. 23850290 10.1016/j.celrep.2013.06.018 PMC4272581

[111] Zhao W, Beers DR, Henkel JS, Zhang W, Urushitani M, Julien JP, et al. (2010). Extracellular mutant SOD1 induces microglial-mediated motoneuron injury. Glia, 58:231-243. 19672969 10.1002/glia.20919 PMC2784168

[112] Deora V, Lee JD, Albornoz EA, McAlary L, Jagaraj CJ, Robertson AAB, et al. (2020). The microglial NLRP3 inflammasome is activated by amyotrophic lateral sclerosis proteins. Glia, 68:407-421. 31596526 10.1002/glia.23728

[113] LaClair KD, Zhou Q, Michaelsen M, Wefers B, Brill MS, Janjic A, et al. (2020). Congenic expression of poly-GA but not poly-PR in mice triggers selective neuron loss and interferon responses found in C9orf72 ALS. Acta Neuropathol, 140:121-142. 32562018 10.1007/s00401-020-02176-0 PMC7360660

[114] Vahsen BF, Gray E, Thompson AG, Ansorge O, Anthony DC, Cowley SA, et al. (2021). Non-neuronal cells in amyotrophic lateral sclerosis — from pathogenesis to biomarkers. Nat Rev Neurol, 17:333-348. 33927394 10.1038/s41582-021-00487-8

[115] Hickman S, Izzy S, Sen P, Morsett L, El Khoury J (2018). Microglia in neurodegeneration. Nat Neurosci, 21:1359-1369. 30258234 10.1038/s41593-018-0242-x PMC6817969

[116] Brandebura AN, Paumier A, Onur TS, Allen NJ (2023). Astrocyte contribution to dysfunction, risk and progression in neurodegenerative disorders. Nat Rev Neurosci, 24:23-39. 36316501 10.1038/s41583-022-00641-1 PMC10198620

[117] Clarke LE, Liddelow SA, Chakraborty C, Münch AE, Heiman M, Barres BA (2018). Normal aging induces A1-like astrocyte reactivity. Proc Natl Acad Sci U S A, 115:E1896-E1905. 29437957 10.1073/pnas.1800165115 PMC5828643

[118] Liddelow SA, Guttenplan KA, Clarke LE, Bennett FC, Bohlen CJ, Schirmer L, et al. (2017). Neurotoxic reactive astrocytes are induced by activated microglia. Nature, 541:481-487. 28099414 10.1038/nature21029 PMC5404890

[119] Guttenplan KA, Weigel MK, Adler DI, Couthouis J, Liddelow SA, Gitler AD, et al. (2020). Knockout of reactive astrocyte activating factors slows disease progression in an ALS mouse model. Nat Commun, 11:1-9. 32719333 10.1038/s41467-020-17514-9 PMC7385161

[120] Gong YH, Parsadanian AS, Andreeva A, Snider WD, Elliott JL (2000). Restricted expression of G86R Cu/Zn superoxide dismutase in astrocytes results in astrocytosis but does not cause motoneuron degeneration. J Neurosci, 20:660-665. 10632595 10.1523/JNEUROSCI.20-02-00660.2000 PMC6772423

[121] Serio A, Bilican B, Barmada SJ, Ando DM, Zhao C, Siller R, et al. (2013). Astrocyte pathology and the absence of non-cell autonomy in an induced pluripotent stem cell model of TDP-43 proteinopathy. Proc Natl Acad Sci U S A, 110:4697-4702. 23401527 10.1073/pnas.1300398110 PMC3607024

[122] Haidet-Phillips AM, Hester ME, Miranda CJ, Meyer K, Braun L, Frakes A, et al. (2011). Astrocytes from familial and sporadic ALS patients are toxic to motor neurons. Nat Biotechnol, 29:824-828. 21832997 10.1038/nbt.1957 PMC3170425

[123] Yamanaka K, Chun SJ, Boillee S, Fujimori-Tonou N, Yamashita H, Gutmann DH, et al. (2008). Astrocytes as determinants of disease progression in inherited amyotrophic lateral sclerosis. Nat Neurosci, 11:251-253. 18246065 10.1038/nn2047 PMC3137510

[124] Wang L, Gutmann DH, Roos RP (2011). Astrocyte loss of mutant SOD1 delays ALS disease onset and progression in G85R transgenic mice. Hum Mol Genet, 20:286-293. 20962037 10.1093/hmg/ddq463

[125] Lepore AC, Rauck B, Dejea C, Pardo AC, Rao MS, Rothstein JD, et al. (2008). Focal transplantation-based astrocyte replacement is neuroprotective in a model of motor neuron disease. Nat Neurosci, 11:1294-1301. 18931666 10.1038/nn.2210 PMC2656686

[126] Papadeas ST, Kraig SE, O’Banion C, Lepore AC, Maragakis NJ (2011). Astrocytes carrying the superoxide dismutase 1 (SOD1 G93A) mutation induce wild-type motor neuron degeneration in vivo. Proc Natl Acad Sci U S A, 108:17803-17808. 21969586 10.1073/pnas.1103141108 PMC3203804

[127] Nagai M, Re DB, Nagata T, Chalazonitis A, Jessell TM, Wichterle H, et al. (2007). Astrocytes expressing ALS-linked mutated SOD1 release factors selectively toxic to motor neurons. Nat Neurosci, 10:615-622. 17435755 10.1038/nn1876 PMC3799799

[128] Endo F, Komine O, Fujimori-Tonou N, Katsuno M, Jin S, Watanabe S, et al. (2015). Astrocyte-Derived TGF-β1 Accelerates Disease Progression in ALS Mice by Interfering with the Neuroprotective Functions of Microglia and T Cells. Cell Rep, 11:592-604. 25892237 10.1016/j.celrep.2015.03.053

[129] Allen SP, Hall B, Castelli LM, Francis L, Woof R, Siskos AP, et al. (2019). Astrocyte adenosine deaminase loss increases motor neuron toxicity in amyotrophic lateral sclerosis. Brain, 142:586-605. 30698736 10.1093/brain/awy353 PMC6391613

[130] Allen SP, Hall B, Woof R, Francis L, Gatto N, Shaw AC, et al. (2019). C9orf72 expansion within astrocytes reduces metabolic flexibility in amyotrophic lateral sclerosis. Brain, 142:3771-3790. 31647549 10.1093/brain/awz302 PMC6906594

[131] Huang C, Huang B, Bi F, Yan LH, Tong J, Huang J, et al. (2014). Profiling the genes affected by pathogenic TDP-43 in astrocytes. J Neurochem, 129:932-939. 24447103 10.1111/jnc.12660 PMC4066372

[132] Tong J, Huang C, Bi F, Wu Q, Huang B, Liu X, et al. (2013). Expression of ALS-linked TDP-43 mutant in astrocytes causes non-cell-autonomous motor neuron death in rats. EMBO J, 32:1917-1926. 23714777 10.1038/emboj.2013.122 PMC3981181

[133] Birger A, Ben-Dor I, Ottolenghi M, Turetsky T, Gil Y, Sweetat S, et al. (2019). Human iPSC-derived astrocytes from ALS patients with mutated C9ORF72 show increased oxidative stress and neurotoxicity. EBioMedicine, 50:274-289. 31787569 10.1016/j.ebiom.2019.11.026 PMC6921360

[134] Greenhalgh AD, David S, Bennett FC (2020). Immune cell regulation of glia during CNS injury and disease. Nat Rev Neurosci, 21:139-152. 32042145 10.1038/s41583-020-0263-9

[135] Lewandowski SA, Nilsson I, Fredriksson L, Lönnerberg P, Muhl L, Zeitelhofer M, et al. (2016). Presymptomatic activation of the PDGF-CC pathway accelerates onset of ALS neurodegeneration. Acta Neuropathol, 131:453-464. 26687981 10.1007/s00401-015-1520-2 PMC4839168

[136] Zhong Z, Deane R, Ali Z, Parisi M, Shapovalov Y, O’Banion MK, et al. (2008). ALS-causing SOD1 mutants generate vascular changes prior to motor neuron degeneration. Nat Neurosci, 11:420-422. 18344992 10.1038/nn2073 PMC2895310

[137] Saul J, Hutchins E, Reiman R, Saul M, Ostrow LW, Harris BT, et al. (2020). Global alterations to the choroid plexus blood-CSF barrier in amyotrophic lateral sclerosis. 9:1-21. 10.1186/s40478-020-00968-9 PMC7318439 32586411

[138] Månberg A, Skene N, Sanders F, Trusohamn M, Remnestål J, Szczepińska A, et al. (2021). Altered perivascular fibroblast activity precedes ALS disease onset. Nat Med. doi: 10.1038/s41591-021-01295-9. PMC7613336 33859435

[139] Butovsky O, Siddiqui S, Gabriely G, Lanser AJ, Dake B, Murugaiyan G, et al. (2012). Modulating inflammatory monocytes with a unique microRNA gene signature ameliorates murine ALS. J Clin Invest, 122:3063-3087. 22863620 10.1172/JCI62636 PMC3428086

[140] Henkel JS, Beers DR, Siklós L, Appel SH (2006). The chemokine MCP-1 and the dendritic and myeloid cells it attracts are increased in the mSOD1 mouse model of ALS. Mol Cell Neurosci, 31:427-437. 16337133 10.1016/j.mcn.2005.10.016

[141] Chiot A, Zaïdi S, Iltis C, Ribon M, Berriat F, Schiaffino L, et al. (2020). Modifying macrophages at the periphery has the capacity to change microglial reactivity and to extend ALS survival. Nat Neurosci, 23:1339-1351. 33077946 10.1038/s41593-020-00718-z

[142] Zondler L, Müller K, Khalaji S, Bliederhäuser C, Ruf WP, Grozdanov V, et al. (2016). Peripheral monocytes are functionally altered and invade the CNS in ALS patients. Acta Neuropathol, 132:391-411. 26910103 10.1007/s00401-016-1548-y

[143] Mrdjen D, Pavlovic A, Hartmann FJ, Schreiner B, Utz SG, Leung BP, et al. (2018). High-Dimensional Single-Cell Mapping of Central Nervous System Immune Cells Reveals Distinct Myeloid Subsets in Health, Aging, and Disease. Immunity, 48:380-395.e6. 29426702 10.1016/j.immuni.2018.01.011

[144] Garofalo S, Cocozza G, Porzia A, Inghilleri M, Scavizzi F, Aronica E, et al. (2020). Natural killer cells modulate motor neuron-immune cell cross talk in models of Amyotrophic Lateral Sclerosis. Nat Commun. doi: 10.1038/s41467-020-15644-8. PMC7156729 32286313

[145] Du Y, Zhao W, Thonhoff JR, Wang J, Wen S, Appel SH (2020). Increased activation ability of monocytes from ALS patients. Exp Neurol, 328:113259. 32105709 10.1016/j.expneurol.2020.113259

[146] Wolfe H, Minogue AM, Rooney S, Lynch MA (2018). Infiltrating macrophages contribute to age-related neuroinflammation in C57/BL6 mice. Mech Ageing Dev, 173:84-91. 29758231 10.1016/j.mad.2018.05.003

[147] Haan N, Zhu B, Wang J, Wei X, Song B (2015). Crosstalk between macrophages and astrocytes affects proliferation, reactive phenotype and inflammatory response, suggesting a role during reactive gliosis following spinal cord injury. J Neuroinflammation, 12:1-10. 10.1186/s12974-015-0327-3 PMC4457974 26025034

[148] Chiu IM, Phatnani H, Kuligowski M, Tapia JC, Carrasco MA, Zhang M, et al. (2009). Activation of innate and humoral immunity in the peripheral nervous system of ALS transgenic mice. Proc Natl Acad Sci, 106:20960-20965. 19933335 10.1073/pnas.0911405106 PMC2791631

[149] Greenhalgh AD, Zarruk JG, Healy LM, Baskar Jesudasan SJ, Jhelum P, Salmon CK, et al. (2018). Peripherally derived macrophages modulate microglial function to reduce inflammation after CNS injury. PLoS Biol, 16:1-29. 10.1371/journal.pbio.2005264 PMC6205650 30332405

[150] Nardo G, Trolese MC, Vito G De, Cecchi R, Riva N, Dina G, et al. (2016). Immune response in peripheral axons delays disease progression in SOD1 G93A mice. J Neuroinflammation, 1-16. 27717377 10.1186/s12974-016-0732-2 PMC5055725

[151] Murdock BJ, Zhou T, Kashlan SR, Little RJ, Goutman SA, Feldman EL (2017). Correlation of Peripheral Immunity With Rapid Amyotrophic Lateral Sclerosis Progression. JAMA Neurol, 74:1446. 28973548 10.1001/jamaneurol.2017.2255 PMC5822195

[152] Beers DR, Henkel JS, Zhao W, Wang J, Appel SH (2008). CD4+ T cells support glial neuroprotection, slow disease progression, and modify glial morphology in an animal model of inherited ALS. Proc Natl Acad Sci, 105:15558-15563. 18809917 10.1073/pnas.0807419105 PMC2547419

[153] Chiu IM, Chen A, Zheng Y, Kosaras B, Tsiftsoglou SA, Vartanian TK, et al. (2008). T lymphocytes potentiate endogenous neuroprotective inflammation in a mouse model of ALS. Proc Natl Acad Sci U S A, 105:17913-17918. 18997009 10.1073/pnas.0804610105 PMC2581614

[154] Zhao W, Beers DR, Liao B, Henkel JS, Appel SH (2012). Regulatory T lymphocytes from ALS mice suppress microglia and effector T lymphocytes through different cytokine-mediated mechanisms. Neurobiol Dis, 48:418-428. 22820142 10.1016/j.nbd.2012.07.008 PMC3897268

[155] Beers DR, Henkel JS, Zhao W, Wang J, Huang A, Wen S, et al. (2011). Endogenous regulatory T lymphocytes ameliorate amyotrophic lateral sclerosis in mice and correlate with disease progression in patients with amyotrophic lateral sclerosis. Brain, 134:1293-1314. 21596768 10.1093/brain/awr074 PMC3097891

[156] Sheean RK, McKay FC, Cretney E, Bye CR, Perera ND, Tomas D, et al. (2018). Association of regulatory T-Cell Expansion with progression of amyotrophic lateral sclerosis a study of humans and a transgenic mouse model. JAMA Neurol, 75:681-689. 29507931 10.1001/jamaneurol.2018.0035 PMC5885208

[157] Henkel JS, Beers DR, Wen S, Rivera AL, Toennis KM, Appel JE, et al. (2013). Regulatory T-lymphocytes mediate amyotrophic lateral sclerosis progression and survival. EMBO Mol Med, 5:64-79. 23143995 10.1002/emmm.201201544 PMC3569654

[158] Thonhoff JR, Beers DR, Zhao W, Pleitez M, Simpson EP, Berry JD, et al. (2018). Expanded autologous regulatory T-lymphocyte infusions in ALS A phase I, first-in-human study. Neurol Neuroimmunol NeuroInflammation. doi: 10.1212/NXI.0000000000000465. PMC5961523 29845093

[159] Arbour D, Vande Velde C, Robitaille R (2017). New perspectives on amyotrophic lateral sclerosis: the role of glial cells at the neuromuscular junction. J Physiol, 595:647-661. 27633977 10.1113/JP270213 PMC5285712

[160] Nardo G, Trolese MC, Verderio M, Mariani A, De Paola M, Riva N, et al. (2018). Counteracting roles of MHCI and CD8+ T cells in the peripheral and central nervous system of ALS SOD1G93A mice. Mol Neurodegener, 13:1-24. 30092791 10.1186/s13024-018-0271-7 PMC6085701

[161] Cui C, Ingre C, Yin L, Li X, Andersson J, Seitz C, et al. (2022). Correlation between leukocyte phenotypes and prognosis of amyotrophic lateral sclerosis. Elife, 11:1-16. 10.7554/eLife.74065 PMC8923665 35287794

[162] Murdock BJ, Famie JP, Piecuch CE, Raue KD, Mendelson FE, Pieroni CH, et al. (2021). Natural killer cells associate with amyotrophic lateral sclersois in a sex- and age-dependent manner. JCI Insight, 6:1-15. 10.1172/jci.insight.147129 PMC8262328 33974561

[163] Figueroa-Romero C, Monteagudo A, Murdock BJ, Famie JP, Webber-Davis IF, Piecuch CE, et al. (2022). Tofacitinib Suppresses Natural Killer Cells In Vitro and In Vivo: Implications for Amyotrophic Lateral Sclerosis. Front Immunol, 13:1-15. 10.3389/fimmu.2022.773288 PMC8859451 35197969

[164] Lanier LL (2005). NK cell recognition. Annu Rev Immunol, 23:225-74. 15771571 10.1146/annurev.immunol.23.021704.115526

[165] Song S, Miranda CJ, Braun L, Meyer K, Frakes AE, Ferraiuolo L, et al. (2016). Major histocompatibility complex class I molecules protect motor neurons from astrocyte-induced toxicity in amyotrophic lateral sclerosis. Nat Med, 1-10. 26928464 10.1038/nm.4052 PMC4823173

[166] Mead RJ, Shan N, Reiser HJ, Marshall F, Shaw PJ (2023). Amyotrophic lateral sclerosis: a neurodegenerative disorder poised for successful therapeutic translation. Nat Rev Drug Discov, 22:185-212. 36543887 10.1038/s41573-022-00612-2 PMC9768794

[167] Miller T, Cudkowicz M, Shaw PJ, Andersen PM, Atassi N, Bucelli RC, et al. (2020). Phase 1-2 Trial of Antisense Oligonucleotide Tofersen for SOD1 ALS. N Engl J Med, 383:109-119. 32640130 10.1056/NEJMoa2003715

[168] Paganoni S, Hendrix S, Dickson SP, Knowlton N, Macklin EA, Berry JD, et al. (2021). Long-term survival of participants in the CENTAUR trial of sodium phenylbutyrate-taurursodiol in amyotrophic lateral sclerosis. Muscle and Nerve, 63:31-39. 33063909 10.1002/mus.27091 PMC7820979

[169] Paganoni S, Macklin EA, Hendrix S, Berry JD, Elliott MA, Maiser S, et al. (2020). Trial of Sodium Phenylbutyrate-Taurursodiol for Amyotrophic Lateral Sclerosis. N Engl J Med, 383:919-930. 32877582 10.1056/NEJMoa1916945 PMC9134321

[170] Giovannelli I, Higginbottom A, Kirby J, Azzouz M, Shaw PJ (2023). Prospects for gene replacement therapies in amyotrophic lateral sclerosis. Nat Rev Neurol, 19:39-52. 36481799 10.1038/s41582-022-00751-5

[171] Mendell JR, Al-Zaidy S, Shell R, Arnold WD, Rodino-Klapac LR, Prior TW, et al. (2017). Single-Dose Gene-Replacement Therapy for Spinal Muscular Atrophy. N Engl J Med, 377:1713-1722. 29091557 10.1056/NEJMoa1706198

[172] Korobeynikov VA, Lyashchenko AK, Blanco-Redondo B, Jafar-Nejad P, Shneider NA (2022). Antisense oligonucleotide silencing of FUS expression as a therapeutic approach in amyotrophic lateral sclerosis. Nat Med, 28:104-116. 35075293 10.1038/s41591-021-01615-z PMC8799464

[173] Tran H, Moazami MP, Yang H, McKenna-Yasek D, Douthwright CL, Pinto C, et al. (2022). Suppression of mutant C9orf72 expression by a potent mixed backbone antisense oligonucleotide. Nat Med, 28:117-124. 34949835 10.1038/s41591-021-01557-6 PMC8861976

[174] McCampbell A, Cole T, Wegener AJ, Tomassy GS, Setnicka A, Farley BJ, et al. (2018). Antisense oligonucleotides extend survival and reverse decrement in muscle response in ALS models. J Clin Invest, 128:3558-3567. 30010620 10.1172/JCI99081 PMC6063493

[175] Becker LA, Huang B, Bieri G, Ma R, Knowles DA, Jafar-Nejad P, et al. (2017). Therapeutic reduction of ataxin-2 extends lifespan and reduces pathology in TDP-43 mice. Nature, 544:367-371. 28405022 10.1038/nature22038 PMC5642042

[176] Freischmidt A, Wieland T, Richter B, Ruf W, Schaeffer V, Müller K, et al. (2015). Haploinsufficiency of TBK1 causes familial ALS and fronto-temporal dementia. Nat Neurosci, 18:631-636. 25803835 10.1038/nn.4000

[177] Zhou R, Zhang Q, Xu P (2020). TBK1, a central kinase in innate immune sensing of nucleic acids and beyond. Acta Biochim Biophys Sin (Shanghai), 52:757-767. 32458982 10.1093/abbs/gmaa051

[178] de Majo M, Topp SD, Smith BN, Nishimura AL, Chen HJ, Gkazi AS, et al. (2018). ALS-associated missense and nonsense TBK1 mutations can both cause loss of kinase function. Neurobiol Aging, 71:266.e1-266.e10. 10.1016/j.neurobiolaging.2018.06.015 PMC6983933 30033073

[179] Ye J, Cheung J, Gerbino V, Ahlsén G, Zimanyi C, Hirsh D, et al. (2019). Effects of ALS-associated TANK binding kinase 1 mutations on protein-protein interactions and kinase activity. Proc Natl Acad Sci U S A, 116:24517-24526. 31748271 10.1073/pnas.1915732116 PMC6900539

[180] Duan W, Guo M, Yi L, Zhang J, Bi Y, Liu Y, et al. (2019). Deletion of Tbk1 disrupts autophagy and reproduces behavioral and locomotor symptoms of FTD-ALS in mice. Aging (Albany NY), 11:2457-2476. 31039129 10.18632/aging.101936 PMC6519994

[181] Akizuki M, Yamashita H, Uemura K, Maruyama H, Kawakami H, Ito H, et al. (2013). Optineurin suppression causes neuronal cell death via NF-κB pathway. J Neurochem, 126:699-704. 23721573 10.1111/jnc.12326

[182] Kishikawa H, Wu D, Hu GF (2008). Targeting angiogenin in therapy of amyotropic lateral sclerosis. Expert Opin Ther Targets, 12:1229-1242. 18781822 10.1517/14728222.12.10.1229 PMC2782700

[183] Hudry E, Vandenberghe LH (2019). Therapeutic AAV Gene Transfer to the Nervous System: A Clinical Reality. Neuron, 101:839-862. 30844402 10.1016/j.neuron.2019.02.017 PMC11804970

[184] Fournier CN, Schoenfeld D, Berry JD, Cudkowicz ME, Chan J, Quinn C, et al. (2018). An open label study of a novel immunosuppression intervention for the treatment of amyotrophic lateral sclerosis. Amyotroph Lateral Scler Front Degener, 19:242-249. 10.1080/21678421.2017.1421666 29308669

[185] Gotkine M, Caraco Y, Lerner Y, Blotnick S, Wanounou M, Slutsky SG, et al. (2023). Safety and efficacy of first-in-man intrathecal injection of human astrocytes (AstroRx®) in ALS patients: phase I/IIa clinical trial results. J Transl Med, 21:1-12. 36788520 10.1186/s12967-023-03903-3 PMC9927047

[186] Baloh RH, Johnson JP, Avalos P, Allred P, Svendsen S, Gowing G, et al. (2022). Transplantation of human neural progenitor cells secreting GDNF into the spinal cord of patients with ALS: a phase 1/2a trial. Nat Med, 28:1813-1822. 36064599 10.1038/s41591-022-01956-3 PMC9499868

[187] Berry JD, Cudkowicz ME, Windebank AJ, Staff NP, Owegi M, Nicholson K, et al. (2019). NurOwn, phase 2, randomized, clinical trial in patients with ALS: Safety, clinical, and biomarker results. Neurology, 93:E2294-E2305. 31740545 10.1212/WNL.0000000000008620 PMC6937497

[188] Cudkowicz ME, Lindborg SR, Goyal NA, Miller RG, Burford MJ, Berry JD, et al. (2022). A randomized <scp>placebo-controlled</scp> phase 3 study of mesenchymal stem cells induced to secrete high levels of neurotrophic factors in amyotrophic lateral sclerosis. Muscle Nerve, 65:291-302. 34890069 10.1002/mus.27472 PMC9305113

[189] Camu W, Mickunas M, Veyrune JL, Payan C, Garlanda C, Locati M, et al. (2020). Repeated 5-day cycles of low dose aldesleukin in amyotrophic lateral sclerosis (IMODALS): A phase 2a randomised, double-blind, placebo-controlled trial. EBioMedicine. doi: 10.1016/j.ebiom.2020.102844. PMC7502670 32651161

[190] Vallarola A, Sironi F, Tortarolo M, Gatto N, De Gioia R, Pasetto L, et al. (2018). RNS60 exerts therapeutic effects in the SOD1 ALS mouse model through protective glia and peripheral nerve rescue. J Neuroinflammation, 15:1-22. 29495962 10.1186/s12974-018-1101-0 PMC5833072

[191] Beghi E, Pupillo E, Bianchi E, Bonetto V, Luotti S, Pasetto L, et al. (2023). Effect of RNS60 in amyotrophic lateral sclerosis: a phase II multicentre, randomized, double-blind, placebo-controlled trial. Eur J Neurol, 30:69-86. 36148821 10.1111/ene.15573 PMC10092300

[192] Ketabforoush AHME, Chegini R, Barati S, Tahmasebi F, Moghisseh B, Joghataei MT, et al. (2023). Masitinib: The promising actor in the next season of the Amyotrophic Lateral Sclerosis treatment series. Biomed Pharmacother, 160:114378. 36774721 10.1016/j.biopha.2023.114378

[193] Mora JS, Bradley WG, Chaverri D, Hernández-Barral M, Mascias J, Gamez J, et al. (2021). Long-term survival analysis of masitinib in amyotrophic lateral sclerosis. Ther Adv Neurol Disord, 14:175628642110303. 10.1177/17562864211030365 PMC8388186 34457038

[194] Mora JS, Genge A, Chio A, Estol CJ, Chaverri D, Hernández M, et al. (2020). Masitinib as an add-on therapy to riluzole in patients with amyotrophic lateral sclerosis: a randomized clinical trial. Amyotroph Lateral Scler Front Degener, 21:5-14. 10.1080/21678421.2019.1632346 31280619

[195] Oskarsson B, Maragakis N, Bedlack RS, Goyal N, Meyer JA, Genge A, et al. (2021). MN-166 (ibudilast) in amyotrophic lateral sclerosis in a Phase IIb/III study: COMBAT-ALS study design. Neurodegener Dis Manag, 11:431-443. 34816762 10.2217/nmt-2021-0042

[196] Tönges L, Günther R, Suhr M, Jansen J, Balck A, Saal KA, et al. (2014). Rho kinase inhibition modulates microglia activation and improves survival in a model of amyotrophic lateral sclerosis. Glia, 62:217-232. 24311453 10.1002/glia.22601

